# Search for new physics in top quark production in dilepton final states in proton-proton collisions at $\sqrt{s} = 13\,\text {TeV}$

**DOI:** 10.1140/epjc/s10052-019-7387-y

**Published:** 2019-11-01

**Authors:** A. M. Sirunyan, A. Tumasyan, W. Adam, F. Ambrogi, E. Asilar, T. Bergauer, J. Brandstetter, M. Dragicevic, J. Erö, A. Escalante Del Valle, M. Flechl, R. Frühwirth, V. M. Ghete, J. Hrubec, M. Jeitler, N. Krammer, I. Krätschmer, D. Liko, T. Madlener, I. Mikulec, N. Rad, H. Rohringer, J. Schieck, R. Schöfbeck, M. Spanring, D. Spitzbart, W. Waltenberger, J. Wittmann, C.-E. Wulz, M. Zarucki, V. Chekhovsky, V. Mossolov, J. Suarez Gonzalez, E. A. De Wolf, D. Di Croce, X. Janssen, J. Lauwers, A. Lelek, M. Pieters, H. Van Haevermaet, P. Van Mechelen, N. Van Remortel, S. Abu Zeid, F. Blekman, J. D’Hondt, J. De Clercq, K. Deroover, G. Flouris, D. Lontkovskyi, S. Lowette, I. Marchesini, S. Moortgat, L. Moreels, Q. Python, K. Skovpen, S. Tavernier, W. Van Doninck, P. Van Mulders, I. Van Parijs, D. Beghin, B. Bilin, H. Brun, B. Clerbaux, G. De Lentdecker, H. Delannoy, B. Dorney, G. Fasanella, L. Favart, A. Grebenyuk, A. K. Kalsi, T. Lenzi, J. Luetic, N. Postiau, E. Starling, L. Thomas, C. Vander Velde, P. Vanlaer, D. Vannerom, Q. Wang, T. Cornelis, D. Dobur, A. Fagot, M. Gul, I. Khvastunov, D. Poyraz, C. Roskas, D. Trocino, M. Tytgat, W. Verbeke, B. Vermassen, M. Vit, N. Zaganidis, H. Bakhshiansohi, O. Bondu, G. Bruno, C. Caputo, P. David, C. Delaere, M. Delcourt, A. Giammanco, G. Krintiras, V. Lemaitre, A. Magitteri, K. Piotrzkowski, A. Saggio, M. Vidal Marono, P. Vischia, J. Zobec, F. L. Alves, G. A. Alves, G. Correia Silva, C. Hensel, A. Moraes, M. E. Pol, P. Rebello Teles, E. Belchior Batista Das Chagas, W. Carvalho, J. Chinellato, E. Coelho, E. M. Da Costa, G. G. Da Silveira, D. De Jesus Damiao, C. De Oliveira Martins, S. Fonseca De Souza, H. Malbouisson, D. Matos Figueiredo, M. Melo De Almeida, C. Mora Herrera, L. Mundim, H. Nogima, W. L. Prado Da Silva, L. J. Sanchez Rosas, A. Santoro, A. Sznajder, M. Thiel, E. J. Tonelli Manganote, F. Torres Da Silva De Araujo, A. Vilela Pereira, S. Ahuja, C. A. Bernardes, L. Calligaris, T. R. Fernandez Perez Tomei, E. M. Gregores, P. G. Mercadante, S. F. Novaes, Sandra S. Padula, A. Aleksandrov, R. Hadjiiska, P. Iaydjiev, A. Marinov, M. Misheva, M. Rodozov, M. Shopova, G. Sultanov, A. Dimitrov, L. Litov, B. Pavlov, P. Petkov, W. Fang, X. Gao, L. Yuan, M. Ahmad, J. G. Bian, G. M. Chen, H. S. Chen, M. Chen, Y. Chen, C. H. Jiang, D. Leggat, H. Liao, Z. Liu, S. M. Shaheen, A. Spiezia, J. Tao, E. Yazgan, H. Zhang, S. Zhang, J. Zhao, Y. Ban, G. Chen, A. Levin, J. Li, L. Li, Q. Li, Y. Mao, S. J. Qian, D. Wang, Y. Wang, C. Avila, A. Cabrera, C. A. Carrillo Montoya, L. F. Chaparro Sierra, C. Florez, C. F. González Hernández, M. A. Segura Delgado, B. Courbon, N. Godinovic, D. Lelas, I. Puljak, T. Sculac, Z. Antunovic, M. Kovac, V. Brigljevic, D. Ferencek, K. Kadija, B. Mesic, M. Roguljic, A. Starodumov, T. Susa, M. W. Ather, A. Attikis, M. Kolosova, G. Mavromanolakis, J. Mousa, C. Nicolaou, F. Ptochos, P. A. Razis, H. Rykaczewski, M. Finger, M. Finger, E. Ayala, E. Carrera Jarrin, H. Abdalla, A. Mohamedj, E. Salama, S. Bhowmik, A. Carvalho Antunes De Oliveira, R. K. Dewanjee, K. Ehataht, M. Kadastik, M. Raidal, C. Veelken, P. Eerola, H. Kirschenmann, J. Pekkanen, M. Voutilainen, J. Havukainen, J. K. Heikkilä, T. Järvinen, V. Karimäki, R. Kinnunen, T. Lampén, K. Lassila-Perini, S. Laurila, S. Lehti, T. Lindén, P. Luukka, T. Mäenpää, H. Siikonen, E. Tuominen, J. Tuominiemi, T. Tuuva, M. Besancon, F. Couderc, M. Dejardin, D. Denegri, J. L. Faure, F. Ferri, S. Ganjour, A. Givernaud, P. Gras, G. Hamel de Monchenault, P. Jarry, C. Leloup, E. Locci, J. Malcles, G. Negro, J. Rander, A. Rosowsky, M. Ö. Sahin, M. Titov, A. Abdulsalam, C. Amendola, I. Antropov, F. Beaudette, P. Busson, C. Charlot, R. Granier de Cassagnac, I. Kucher, A. Lobanov, J. Martin Blanco, C. Martin Perez, M. Nguyen, C. Ochando, G. Ortona, P. Paganini, J. Rembser, R. Salerno, J. B. Sauvan, Y. Sirois, A. G. Stahl Leiton, A. Zabi, A. Zghiche, J.-L. Agram, J. Andrea, D. Bloch, G. Bourgatte, J.-M. Brom, E. C. Chabert, V. Cherepanov, C. Collard, E. Conte, J.-C. Fontaine, D. Gelé, U. Goerlach, M. Jansová, A.-C. Le Bihan, N. Tonon, P. Van Hove, S. Gadrat, S. Beauceron, C. Bernet, G. Boudoul, N. Chanon, R. Chierici, D. Contardo, P. Depasse, H. El Mamouni, J. Fay, L. Finco, S. Gascon, M. Gouzevitch, G. Grenier, B. Ille, F. Lagarde, I. B. Laktineh, H. Lattaud, M. Lethuillier, L. Mirabito, S. Perries, A. Popov, V. Sordini, G. Touquet, M. Vander Donckt, S. Viret, T. Toriashvili, Z. Tsamalaidze, C. Autermann, L. Feld, M. K. Kiesel, K. Klein, M. Lipinski, M. Preuten, M. P. Rauch, C. Schomakers, J. Schulz, M. Teroerde, B. Wittmer, A. Albert, M. Erdmann, S. Erdweg, T. Esch, R. Fischer, S. Ghosh, T. Hebbeker, C. Heidemann, K. Hoepfner, H. Keller, L. Mastrolorenzo, M. Merschmeyer, A. Meyer, P. Millet, S. Mukherjee, T. Pook, A. Pozdnyakov, M. Radziej, H. Reithler, M. Rieger, A. Schmidt, D. Teyssier, S. Thüer, G. Flügge, O. Hlushchenko, T. Kress, T. Müller, A. Nehrkorn, A. Nowack, C. Pistone, O. Pooth, D. Roy, H. Sert, A. Stahl, M. Aldaya Martin, T. Arndt, C. Asawatangtrakuldee, I. Babounikau, K. Beernaert, O. Behnke, U. Behrens, A. Bermúdez Martínez, D. Bertsche, A. A. Bin Anuar, K. Borras, V. Botta, A. Campbell, P. Connor, C. Contreras-Campana, V. Danilov, A. De Wit, M. M. Defranchis, C. Diez Pardos, D. Domínguez Damiani, G. Eckerlin, T. Eichhorn, A. Elwood, E. Eren, E. Gallo, A. Geiser, J. M. Grados Luyando, A. Grohsjean, M. Guthoff, M. Haranko, A. Harb, H. Jung, M. Kasemann, J. Keaveney, C. Kleinwort, J. Knolle, D. Krücker, W. Lange, T. Lenz, J. Leonard, K. Lipka, W. Lohmann, R. Mankel, I.-A. Melzer-Pellmann, A. B. Meyer, M. Meyer, M. Missiroli, G. Mittag, J. Mnich, V. Myronenko, S. K. Pflitsch, D. Pitzl, A. Raspereza, A. Saibel, M. Savitskyi, P. Saxena, P. Schütze, C. Schwanenberger, R. Shevchenko, A. Singh, H. Tholen, O. Turkot, A. Vagnerini, M. Van De Klundert, G. P. Van Onsem, R. Walsh, Y. Wen, K. Wichmann, C. Wissing, O. Zenaiev, R. Aggleton, S. Bein, L. Benato, A. Benecke, T. Dreyer, A. Ebrahimi, E. Garutti, D. Gonzalez, P. Gunnellini, J. Haller, A. Hinzmann, A. Karavdina, G. Kasieczka, R. Klanner, R. Kogler, N. Kovalchuk, S. Kurz, V. Kutzner, J. Lange, D. Marconi, J. Multhaup, M. Niedziela, C. E. N. Niemeyer, D. Nowatschin, A. Perieanu, A. Reimers, O. Rieger, C. Scharf, P. Schleper, S. Schumann, J. Schwandt, J. Sonneveld, H. Stadie, G. Steinbrück, F. M. Stober, M. Stöver, B. Vormwald, I. Zoi, M. Akbiyik, C. Barth, M. Baselga, S. Baur, E. Butz, R. Caspart, T. Chwalek, F. Colombo, W. De Boer, A. Dierlamm, K. El Morabit, N. Faltermann, B. Freund, M. Giffels, M. A. Harrendorf, F. Hartmann, S. M. Heindl, U. Husemann, I. Katkov, S. Kudella, S. Mitra, M. U. Mozer, Th. Müller, M. Musich, M. Plagge, G. Quast, K. Rabbertz, M. Schröder, I. Shvetsov, H. J. Simonis, R. Ulrich, S. Wayand, M. Weber, T. Weiler, C. Wöhrmann, R. Wolf, G. Anagnostou, G. Daskalakis, T. Geralis, A. Kyriakis, D. Loukas, G. Paspalaki, A. Agapitos, G. Karathanasis, P. Kontaxakis, A. Panagiotou, I. Papavergou, N. Saoulidou, K. Vellidis, K. Kousouris, I. Papakrivopoulos, G. Tsipolitis, I. Evangelou, C. Foudas, P. Gianneios, P. Katsoulis, P. Kokkas, S. Mallios, N. Manthos, I. Papadopoulos, E. Paradas, J. Strologas, F. A. Triantis, D. Tsitsonis, M. Bartók, M. Csanad, N. Filipovic, P. Major, M. I. Nagy, G. Pasztor, O. Surányi, G. I. Veres, G. Bencze, C. Hajdu, D. Horvath, Á. Hunyadi, F. Sikler, T. Á. Vámi, V. Veszpremi, G. Vesztergombi, N. Beni, S. Czellar, J. Karancsi, A. Makovec, J. Molnar, Z. Szillasi, P. Raics, Z. L. Trocsanyi, B. Ujvari, S. Choudhury, J. R. Komaragiri, P. C. Tiwari, S. Bahinipati, C. Kar, P. Mal, K. Mandal, A. Nayak, S. Roy Chowdhury, D. K. Sahoo, S. K. Swain, S. Bansal, S. B. Beri, V. Bhatnagar, S. Chauhan, R. Chawla, N. Dhingra, R. Gupta, A. Kaur, M. Kaur, S. Kaur, P. Kumari, M. Lohan, M. Meena, A. Mehta, K. Sandeep, S. Sharma, J. B. Singh, A. K. Virdi, G. Walia, A. Bhardwaj, B. C. Choudhary, R. B. Garg, M. Gola, S. Keshri, A. Kumar, S. Malhotra, M. Naimuddin, P. Priyanka, K. Ranjan, A. Shah, R. Sharma, R. Bhardwaj, M. Bharti, R. Bhattacharya, S. Bhattacharya, U. Bhawandeep, D. Bhowmik, S. Dey, S. Dutt, S. Dutta, S. Ghosh, M. Maity, K. Mondal, S. Nandan, A. Purohit, P. K. Rout, A. Roy, G. Saha, S. Sarkar, T. Sarkar, M. Sharan, B. Singh, S. Thakur, P. K. Behera, A. Muhammad, R. Chudasama, D. Dutta, V. Jha, V. Kumar, D. K. Mishra, P. K. Netrakanti, L. M. Pant, P. Shukla, P. Suggisetti, T. Aziz, M. A. Bhat, S. Dugad, G. B. Mohanty, N. Sur, R. Verma, S. Banerjee, S. Bhattacharya, S. Chatterjee, P. Das, M. Guchait, Sa. Jain, S. Karmakar, S. Kumar, G. Majumder, K. Mazumdar, N. Sahoo, S. Chauhan, S. Dube, V. Hegde, A. Kapoor, K. Kothekar, S. Pandey, A. Rane, A. Rastogi, S. Sharma, S. Chenarani, E. Eskandari Tadavani, S. M. Etesami, M. Khakzad, M. Mohammadi Najafabadi, M. Naseri, F. Rezaei Hosseinabadi, B. Safarzadeh, M. Zeinali, M. Felcini, M. Grunewald, M. Abbrescia, C. Calabria, A. Colaleo, D. Creanza, L. Cristella, N. De Filippis, M. De Palma, A. Di Florio, F. Errico, L. Fiore, A. Gelmi, G. Iaselli, M. Ince, S. Lezki, G. Maggi, M. Maggi, G. Miniello, S. My, S. Nuzzo, A. Pompili, G. Pugliese, R. Radogna, A. Ranieri, G. Selvaggi, A. Sharma, L. Silvestris, R. Venditti, P. Verwilligen, G. Abbiendi, C. Battilana, D. Bonacorsi, L. Borgonovi, S. Braibant-Giacomelli, R. Campanini, P. Capiluppi, A. Castro, F. R. Cavallo, S. S. Chhibra, G. Codispoti, M. Cuffiani, G. M. Dallavalle, F. Fabbri, A. Fanfani, E. Fontanesi, P. Giacomelli, C. Grandi, L. Guiducci, F. Iemmi, S. Lo Meo, S. Marcellini, G. Masetti, A. Montanari, F. L. Navarria, A. Perrotta, F. Primavera, A. M. Rossi, T. Rovelli, G. P. Siroli, N. Tosi, S. Albergo, A. Di Mattia, R. Potenza, A. Tricomi, C. Tuve, G. Barbagli, K. Chatterjee, V. Ciulli, C. Civinini, R. D’Alessandro, E. Focardi, G. Latino, P. Lenzi, M. Meschini, S. Paoletti, L. Russo, G. Sguazzoni, D. Strom, L. Viliani, L. Benussi, S. Bianco, F. Fabbri, D. Piccolo, F. Ferro, R. Mulargia, E. Robutti, S. Tosi, A. Benaglia, A. Beschi, F. Brivio, V. Ciriolo, S. Di Guida, M. E. Dinardo, S. Fiorendi, S. Gennai, A. Ghezzi, P. Govoni, M. Malberti, S. Malvezzi, D. Menasce, F. Monti, L. Moroni, M. Paganoni, D. Pedrini, S. Ragazzi, T. Tabarelli de Fatis, D. Zuolo, S. Buontempo, N. Cavallo, A. De Iorio, A. Di Crescenzo, F. Fabozzi, F. Fienga, G. Galati, A. O. M. Iorio, L. Lista, S. Meola, P. Paolucci, C. Sciacca, E. Voevodina, P. Azzi, N. Bacchetta, D. Bisello, A. Boletti, A. Bragagnolo, R. Carlin, P. Checchia, M. Dall’Osso, P. De Castro Manzano, T. Dorigo, U. Dosselli, F. Gasparini, U. Gasparini, A. Gozzelino, S. Y. Hoh, S. Lacaprara, P. Lujan, M. Margoni, A. T. Meneguzzo, J. Pazzini, M. Presilla, P. Ronchese, R. Rossin, F. Simonetto, A. Tiko, E. Torassa, M. Tosi, M. Zanetti, P. Zotto, G. Zumerle, A. Braghieri, A. Magnani, P. Montagna, S. P. Ratti, V. Re, M. Ressegotti, C. Riccardi, P. Salvini, I. Vai, P. Vitulo, M. Biasini, G. M. Bilei, C. Cecchi, D. Ciangottini, L. Fanò, P. Lariccia, R. Leonardi, E. Manoni, G. Mantovani, V. Mariani, M. Menichelli, A. Rossi, A. Santocchia, D. Spiga, K. Androsov, P. Azzurri, G. Bagliesi, L. Bianchini, T. Boccali, L. Borrello, R. Castaldi, M. A. Ciocci, R. Dell’Orso, G. Fedi, F. Fiori, L. Giannini, A. Giassi, M. T. Grippo, F. Ligabue, E. Manca, G. Mandorli, A. Messineo, F. Palla, A. Rizzi, G. Rolandi, P. Spagnolo, R. Tenchini, G. Tonelli, A. Venturi, P. G. Verdini, L. Barone, F. Cavallari, M. Cipriani, D. Del Re, E. Di Marco, M. Diemoz, S. Gelli, E. Longo, B. Marzocchi, P. Meridiani, G. Organtini, F. Pandolfi, R. Paramatti, F. Preiato, S. Rahatlou, C. Rovelli, F. Santanastasio, N. Amapane, R. Arcidiacono, S. Argiro, M. Arneodo, N. Bartosik, R. Bellan, C. Biino, A. Cappati, N. Cartiglia, F. Cenna, S. Cometti, M. Costa, R. Covarelli, N. Demaria, B. Kiani, C. Mariotti, S. Maselli, E. Migliore, V. Monaco, E. Monteil, M. Monteno, M. M. Obertino, L. Pacher, N. Pastrone, M. Pelliccioni, G. L. Pinna Angioni, A. Romero, M. Ruspa, R. Sacchi, R. Salvatico, K. Shchelina, V. Sola, A. Solano, D. Soldi, A. Staiano, S. Belforte, V. Candelise, M. Casarsa, F. Cossutti, A. Da Rold, G. Della Ricca, F. Vazzoler, A. Zanetti, D. H. Kim, G. N. Kim, M. S. Kim, J. Lee, S. Lee, S. W. Lee, C. S. Moon, Y. D. Oh, S. I. Pak, S. Sekmen, D. C. Son, Y. C. Yang, H. Kim, D. H. Moon, G. Oh, B. Francois, J. Goh, T. J. Kim, S. Cho, S. Choi, Y. Go, D. Gyun, S. Ha, B. Hong, Y. Jo, K. Lee, K. S. Lee, S. Lee, J. Lim, S. K. Park, Y. Roh, H. S. Kim, J. Almond, J. Kim, J. S. Kim, H. Lee, K. Lee, K. Nam, S. B. Oh, B. C. Radburn-Smith, S. h. Seo, U. K. Yang, H. D. Yoo, G. B. Yu, D. Jeon, H. Kim, J. H. Kim, J. S. H. Lee, I. C. Park, Y. Choi, C. Hwang, J. Lee, I. Yu, V. Veckalns, V. Dudenas, A. Juodagalvis, J. Vaitkus, Z. A. Ibrahim, M. A. B. Md Ali, F. Mohamad Idris, W. A. T. Wan Abdullah, M. N. Yusli, Z. Zolkapli, J. F. Benitez, A. Castaneda Hernandez, J. A. Murillo Quijada, H. Castilla-Valdez, E. De La Cruz-Burelo, M. C. Duran-Osuna, I. Heredia-De La Cruz, R. Lopez-Fernandez, J. Mejia Guisao, R. I. Rabadan-Trejo, M. Ramirez-Garcia, G. Ramirez-Sanchez, R. Reyes-Almanza, A. Sanchez-Hernandez, S. Carrillo Moreno, C. Oropeza Barrera, F. Vazquez Valencia, J. Eysermans, I. Pedraza, H. A. Salazar Ibarguen, C. Uribe Estrada, A. Morelos Pineda, D. Krofcheck, S. Bheesette, P. H. Butler, A. Ahmad, M. Ahmad, M. I. Asghar, Q. Hassan, H. R. Hoorani, W. A. Khan, M. A. Shah, M. Shoaib, M. Waqas, H. Bialkowska, M. Bluj, B. Boimska, T. Frueboes, M. Górski, M. Kazana, M. Szleper, P. Traczyk, P. Zalewski, K. Bunkowski, A. Byszuk, K. Doroba, A. Kalinowski, M. Konecki, J. Krolikowski, M. Misiura, M. Olszewski, A. Pyskir, M. Walczak, M. Araujo, P. Bargassa, C. Beirão Da Cruz E Silva, A. Di Francesco, P. Faccioli, B. Galinhas, M. Gallinaro, J. Hollar, N. Leonardo, J. Seixas, G. Strong, O. Toldaiev, J. Varela, S. Afanasiev, P. Bunin, M. Gavrilenko, I. Golutvin, I. Gorbunov, A. Kamenev, V. Karjavine, A. Lanev, A. Malakhov, V. Matveev, P. Moisenz, V. Palichik, V. Perelygin, S. Shmatov, S. Shulha, N. Skatchkov, V. Smirnov, N. Voytishin, A. Zarubin, V. Golovtsov, Y. Ivanov, V. Kim, E. Kuznetsova, P. Levchenko, V. Murzin, V. Oreshkin, I. Smirnov, D. Sosnov, V. Sulimov, L. Uvarov, S. Vavilov, A. Vorobyev, Yu. Andreev, A. Dermenev, S. Gninenko, N. Golubev, A. Karneyeu, M. Kirsanov, N. Krasnikov, A. Pashenkov, A. Shabanov, D. Tlisov, A. Toropin, V. Epshteyn, V. Gavrilov, N. Lychkovskaya, V. Popov, I. Pozdnyakov, G. Safronov, A. Spiridonov, A. Stepennov, V. Stolin, M. Toms, E. Vlasov, A. Zhokin, T. Aushev, R. Chistov, M. Danilov, D. Philippov, E. Tarkovskii, V. Andreev, M. Azarkin, I. Dremin, M. Kirakosyan, A. Terkulov, A. Baskakov, A. Belyaev, E. Boos, V. Bunichev, M. Dubinin, L. Dudko, V. Klyukhin, O. Kodolova, N. Korneeva, I. Lokhtin, S. Obraztsov, M. Perfilov, V. Savrin, A. Barnyakov, V. Blinov, T. Dimova, L. Kardapoltsev, Y. Skovpen, I. Azhgirey, I. Bayshev, S. Bitioukov, V. Kachanov, A. Kalinin, D. Konstantinov, P. Mandrik, V. Petrov, R. Ryutin, S. Slabospitskii, A. Sobol, S. Troshin, N. Tyurin, A. Uzunian, A. Volkov, A. Babaev, S. Baidali, V. Okhotnikov, P. Adzic, P. Cirkovic, D. Devetak, M. Dordevic, P. Milenovic, J. Milosevic, J. Alcaraz Maestre, A. Álvarez Fernández, I. Bachiller, M. Barrio Luna, J. A. Brochero Cifuentes, M. Cerrada, N. Colino, B. De La Cruz, A. Delgado Peris, C. Fernandez Bedoya, J. P. Fernández Ramos, J. Flix, M. C. Fouz, O. Gonzalez Lopez, S. Goy Lopez, J. M. Hernandez, M. I. Josa, D. Moran, A. Pérez-Calero Yzquierdo, J. Puerta Pelayo, I. Redondo, L. Romero, S. Sánchez Navas, M. S. Soares, A. Triossi, C. Albajar, J. F. de Trocóniz, J. Cuevas, C. Erice, J. Fernandez Menendez, S. Folgueras, I. Gonzalez Caballero, J. R. González Fernández, E. Palencia Cortezon, V. Rodríguez Bouza, S. Sanchez Cruz, J. M. Vizan Garcia, I. J. Cabrillo, A. Calderon, B. Chazin Quero, J. Duarte Campderros, M. Fernandez, P. J. Fernández Manteca, A. García Alonso, J. Garcia-Ferrero, G. Gomez, A. Lopez Virto, J. Marco, C. Martinez Rivero, P. Martinez Ruiz del Arbol, F. Matorras, J. Piedra Gomez, C. Prieels, T. Rodrigo, A. Ruiz-Jimeno, L. Scodellaro, N. Trevisani, I. Vila, R. Vilar Cortabitarte, N. Wickramage, D. Abbaneo, B. Akgun, E. Auffray, G. Auzinger, P. Baillon, A. H. Ball, D. Barney, J. Bendavid, M. Bianco, A. Bocci, C. Botta, E. Brondolin, T. Camporesi, M. Cepeda, G. Cerminara, E. Chapon, Y. Chen, G. Cucciati, D. d’Enterria, A. Dabrowski, N. Daci, V. Daponte, A. David, A. De Roeck, N. Deelen, M. Dobson, M. Dünser, N. Dupont, A. Elliott-Peisert, F. Fallavollita, D. Fasanella, G. Franzoni, J. Fulcher, W. Funk, D. Gigi, A. Gilbert, K. Gill, F. Glege, M. Gruchala, M. Guilbaud, D. Gulhan, J. Hegeman, C. Heidegger, V. Innocente, G. M. Innocenti, A. Jafari, P. Janot, O. Karacheban, J. Kieseler, A. Kornmayer, M. Krammer, C. Lange, P. Lecoq, C. Lourenço, L. Malgeri, M. Mannelli, A. Massironi, F. Meijers, J. A. Merlin, S. Mersi, E. Meschi, F. Moortgat, M. Mulders, J. Ngadiuba, S. Nourbakhsh, S. Orfanelli, L. Orsini, F. Pantaleo, L. Pape, E. Perez, M. Peruzzi, A. Petrilli, G. Petrucciani, A. Pfeiffer, M. Pierini, F. M. Pitters, D. Rabady, A. Racz, M. Rovere, H. Sakulin, C. Schäfer, C. Schwick, M. Selvaggi, A. Sharma, P. Silva, P. Sphicas, A. Stakia, J. Steggemann, D. Treille, A. Tsirou, A. Vartak, M. Verzetti, W. D. Zeuner, L. Caminada, K. Deiters, W. Erdmann, R. Horisberger, Q. Ingram, H. C. Kaestli, D. Kotlinski, U. Langenegger, T. Rohe, S. A. Wiederkehr, M. Backhaus, L. Bäni, P. Berger, N. Chernyavskaya, G. Dissertori, M. Dittmar, M. Donegà, C. Dorfer, T. A. Gómez Espinosa, C. Grab, D. Hits, T. Klijnsma, W. Lustermann, R. A. Manzoni, M. Marionneau, M. T. Meinhard, F. Micheli, P. Musella, F. Nessi-Tedaldi, F. Pauss, G. Perrin, L. Perrozzi, S. Pigazzini, M. Reichmann, C. Reissel, D. Ruini, D. A. Sanz Becerra, M. Schönenberger, L. Shchutska, V. R. Tavolaro, K. Theofilatos, M. L. Vesterbacka Olsson, R. Wallny, D. H. Zhu, T. K. Aarrestad, C. Amsler, D. Brzhechko, M. F. Canelli, A. De Cosa, R. Del Burgo, S. Donato, C. Galloni, T. Hreus, B. Kilminster, S. Leontsinis, I. Neutelings, G. Rauco, P. Robmann, D. Salerno, K. Schweiger, C. Seitz, Y. Takahashi, S. Wertz, A. Zucchetta, T. H. Doan, R. Khurana, C. M. Kuo, W. Lin, S. S. Yu, P. Chang, Y. Chao, K. F. Chen, P. H. Chen, W.-S. Hou, Y. F. Liu, R.-S. Lu, E. Paganis, A. Psallidas, A. Steen, B. Asavapibhop, N. Srimanobhas, N. Suwonjandee, A. Bat, F. Boran, S. Cerci, S. Damarseckin, Z. S. Demiroglu, F. Dolek, C. Dozen, I. Dumanoglu, E. Eskut, G. Gokbulut, Y. Guler, E. Gurpinar, I. Hos, C. Isik, E. E. Kangal, O. Kara, A. Kayis Topaksu, U. Kiminsu, M. Oglakci, G. Onengut, K. Ozdemir, A. Polatoz, D. Sunar Cerci, U. G. Tok, S. Turkcapar, I. S. Zorbakir, C. Zorbilmez, B. Isildak, G. Karapinar, M. Yalvac, M. Zeyrek, I. O. Atakisi, E. Gülmez, M. Kaya, O. Kaya, S. Ozkorucuklu, S. Tekten, E. A. Yetkin, M. N. Agaras, A. Cakir, K. Cankocak, Y. Komurcu, S. Sen, B. Grynyov, L. Levchuk, F. Ball, J. J. Brooke, D. Burns, E. Clement, D. Cussans, O. Davignon, H. Flacher, J. Goldstein, G. P. Heath, H. F. Heath, L. Kreczko, D. M. Newbold, S. Paramesvaran, B. Penning, T. Sakuma, D. Smith, V. J. Smith, J. Taylor, A. Titterton, K. W. Bell, A. Belyaev, C. Brew, R. M. Brown, D. Cieri, D. J. A. Cockerill, J. A. Coughlan, K. Harder, S. Harper, J. Linacre, K. Manolopoulos, E. Olaiya, D. Petyt, T. Reis, T. Schuh, C. H. Shepherd-Themistocleous, A. Thea, I. R. Tomalin, T. Williams, W. J. Womersley, R. Bainbridge, P. Bloch, J. Borg, S. Breeze, O. Buchmuller, A. Bundock, D. Colling, P. Dauncey, G. Davies, M. Della Negra, R. Di Maria, P. Everaerts, G. Hall, G. Iles, T. James, M. Komm, C. Laner, L. Lyons, A.-M. Magnan, S. Malik, A. Martelli, J. Nash, A. Nikitenko, V. Palladino, M. Pesaresi, D. M. Raymond, A. Richards, A. Rose, E. Scott, C. Seez, A. Shtipliyski, G. Singh, M. Stoye, T. Strebler, S. Summers, A. Tapper, K. Uchida, T. Virdee, N. Wardle, D. Winterbottom, J. Wright, S. C. Zenz, J. E. Cole, P. R. Hobson, A. Khan, P. Kyberd, C. K. Mackay, A. Morton, I. D. Reid, L. Teodorescu, S. Zahid, K. Call, J. Dittmann, K. Hatakeyama, H. Liu, C. Madrid, B. McMaster, N. Pastika, C. Smith, R. Bartek, A. Dominguez, A. Buccilli, S. I. Cooper, C. Henderson, P. Rumerio, C. West, D. Arcaro, T. Bose, Z. Demiragli, D. Gastler, S. Girgis, D. Pinna, C. Richardson, J. Rohlf, D. Sperka, I. Suarez, L. Sulak, D. Zou, G. Benelli, B. Burkle, X. Coubez, D. Cutts, M. Hadley, J. Hakala, U. Heintz, J. M. Hogan, K. H. M. Kwok, E. Laird, G. Landsberg, J. Lee, Z. Mao, M. Narain, S. Sagir, R. Syarif, E. Usai, D. Yu, R. Band, C. Brainerd, R. Breedon, D. Burns, M. Calderon De La Barca Sanchez, M. Chertok, J. Conway, R. Conway, P. T. Cox, R. Erbacher, C. Flores, G. Funk, W. Ko, O. Kukral, R. Lander, M. Mulhearn, D. Pellett, J. Pilot, S. Shalhout, M. Shi, D. Stolp, D. Taylor, K. Tos, M. Tripathi, Z. Wang, F. Zhang, M. Bachtis, C. Bravo, R. Cousins, A. Dasgupta, S. Erhan, A. Florent, J. Hauser, M. Ignatenko, N. Mccoll, S. Regnard, D. Saltzberg, C. Schnaible, V. Valuev, E. Bouvier, K. Burt, R. Clare, J. W. Gary, S. M. A. Ghiasi Shirazi, G. Hanson, G. Karapostoli, E. Kennedy, F. Lacroix, O. R. Long, M. Olmedo Negrete, M. I. Paneva, W. Si, L. Wang, H. Wei, S. Wimpenny, B. R. Yates, J. G. Branson, P. Chang, S. Cittolin, M. Derdzinski, R. Gerosa, D. Gilbert, B. Hashemi, A. Holzner, D. Klein, G. Kole, V. Krutelyov, J. Letts, M. Masciovecchio, S. May, D. Olivito, S. Padhi, M. Pieri, V. Sharma, M. Tadel, J. Wood, F. Würthwein, A. Yagil, G. Zevi Della Porta, N. Amin, R. Bhandari, C. Campagnari, M. Citron, V. Dutta, M. Franco Sevilla, L. Gouskos, R. Heller, J. Incandela, H. Mei, A. Ovcharova, H. Qu, J. Richman, D. Stuart, S. Wang, J. Yoo, D. Anderson, A. Bornheim, J. M. Lawhorn, N. Lu, H. B. Newman, T. Q. Nguyen, J. Pata, M. Spiropulu, J. R. Vlimant, R. Wilkinson, S. Xie, Z. Zhang, R. Y. Zhu, M. B. Andrews, T. Ferguson, T. Mudholkar, M. Paulini, M. Sun, I. Vorobiev, M. Weinberg, J. P. Cumalat, W. T. Ford, F. Jensen, A. Johnson, E. MacDonald, T. Mulholland, R. Patel, A. Perloff, K. Stenson, K. A. Ulmer, S. R. Wagner, J. Alexander, J. Chaves, Y. Cheng, J. Chu, A. Datta, K. Mcdermott, N. Mirman, J. R. Patterson, D. Quach, A. Rinkevicius, A. Ryd, L. Skinnari, L. Soffi, S. M. Tan, Z. Tao, J. Thom, J. Tucker, P. Wittich, M. Zientek, S. Abdullin, M. Albrow, M. Alyari, G. Apollinari, A. Apresyan, A. Apyan, S. Banerjee, L. A. T. Bauerdick, A. Beretvas, J. Berryhill, P. C. Bhat, K. Burkett, J. N. Butler, A. Canepa, G. B. Cerati, H. W. K. Cheung, F. Chlebana, M. Cremonesi, J. Duarte, V. D. Elvira, J. Freeman, Z. Gecse, E. Gottschalk, L. Gray, D. Green, S. Grünendahl, O. Gutsche, J. Hanlon, R. M. Harris, S. Hasegawa, J. Hirschauer, Z. Hu, B. Jayatilaka, S. Jindariani, M. Johnson, U. Joshi, B. Klima, M. J. Kortelainen, B. Kreis, S. Lammel, D. Lincoln, R. Lipton, M. Liu, T. Liu, J. Lykken, K. Maeshima, J. M. Marraffino, D. Mason, P. McBride, P. Merkel, S. Mrenna, S. Nahn, V. O’Dell, K. Pedro, C. Pena, O. Prokofyev, G. Rakness, F. Ravera, A. Reinsvold, L. Ristori, A. Savoy-Navarro, B. Schneider, E. Sexton-Kennedy, A. Soha, W. J. Spalding, L. Spiegel, S. Stoynev, J. Strait, N. Strobbe, L. Taylor, S. Tkaczyk, N. V. Tran, L. Uplegger, E. W. Vaandering, C. Vernieri, M. Verzocchi, R. Vidal, M. Wang, H. A. Weber, D. Acosta, P. Avery, P. Bortignon, D. Bourilkov, A. Brinkerhoff, L. Cadamuro, A. Carnes, D. Curry, R. D. Field, S. V. Gleyzer, B. M. Joshi, J. Konigsberg, A. Korytov, K. H. Lo, P. Ma, K. Matchev, N. Menendez, G. Mitselmakher, D. Rosenzweig, K. Shi, J. Wang, S. Wang, X. Zuo, Y. R. Joshi, S. Linn, A. Ackert, T. Adams, A. Askew, S. Hagopian, V. Hagopian, K. F. Johnson, T. Kolberg, G. Martinez, T. Perry, H. Prosper, A. Saha, C. Schiber, R. Yohay, M. M. Baarmand, V. Bhopatkar, S. Colafranceschi, M. Hohlmann, D. Noonan, M. Rahmani, T. Roy, M. Saunders, F. Yumiceva, M. R. Adams, L. Apanasevich, D. Berry, R. R. Betts, R. Cavanaugh, X. Chen, S. Dittmer, O. Evdokimov, C. E. Gerber, D. A. Hangal, D. J. Hofman, K. Jung, J. Kamin, C. Mills, M. B. Tonjes, N. Varelas, H. Wang, X. Wang, Z. Wu, J. Zhang, M. Alhusseini, B. Bilki, W. Clarida, K. Dilsiz, S. Durgut, R. P. Gandrajula, M. Haytmyradov, V. Khristenko, J.-P. Merlo, A. Mestvirishvili, A. Moeller, J. Nachtman, H. Ogul, Y. Onel, F. Ozok, A. Penzo, C. Snyder, E. Tiras, J. Wetzel, B. Blumenfeld, A. Cocoros, N. Eminizer, D. Fehling, L. Feng, A. V. Gritsan, W. T. Hung, P. Maksimovic, J. Roskes, U. Sarica, M. Swartz, M. Xiao, A. Al-bataineh, P. Baringer, A. Bean, S. Boren, J. Bowen, A. Bylinkin, J. Castle, S. Khalil, A. Kropivnitskaya, D. Majumder, W. Mcbrayer, M. Murray, C. Rogan, S. Sanders, E. Schmitz, J. D. Tapia Takaki, Q. Wang, S. Duric, A. Ivanov, K. Kaadze, D. Kim, Y. Maravin, D. R. Mendis, T. Mitchell, A. Modak, A. Mohammadi, F. Rebassoo, D. Wright, A. Baden, O. Baron, A. Belloni, S. C. Eno, Y. Feng, C. Ferraioli, N. J. Hadley, S. Jabeen, G. Y. Jeng, R. G. Kellogg, J. Kunkle, A. C. Mignerey, S. Nabili, F. Ricci-Tam, M. Seidel, Y. H. Shin, A. Skuja, S. C. Tonwar, K. Wong, D. Abercrombie, B. Allen, V. Azzolini, A. Baty, R. Bi, S. Brandt, W. Busza, I. A. Cali, M. D’Alfonso, G. Gomez Ceballos, M. Goncharov, P. Harris, D. Hsu, M. Hu, Y. Iiyama, M. Klute, D. Kovalskyi, Y.-J. Lee, P. D. Luckey, B. Maier, A. C. Marini, C. Mcginn, C. Mironov, S. Narayanan, X. Niu, C. Paus, D. Rankin, C. Roland, G. Roland, Z. Shi, G. S. F. Stephans, K. Sumorok, K. Tatar, D. Velicanu, J. Wang, T. W. Wang, B. Wyslouch, A. C. Benvenuti, R. M. Chatterjee, A. Evans, P. Hansen, J. Hiltbrand, Sh. Jain, S. Kalafut, M. Krohn, Y. Kubota, Z. Lesko, J. Mans, R. Rusack, M. A. Wadud, J. G. Acosta, S. Oliveros, E. Avdeeva, K. Bloom, D. R. Claes, C. Fangmeier, F. Golf, R. Gonzalez Suarez, R. Kamalieddin, I. Kravchenko, J. Monroy, J. E. Siado, G. R. Snow, B. Stieger, A. Godshalk, C. Harrington, I. Iashvili, A. Kharchilava, C. Mclean, D. Nguyen, A. Parker, S. Rappoccio, B. Roozbahani, G. Alverson, E. Barberis, C. Freer, Y. Haddad, A. Hortiangtham, G. Madigan, D. M. Morse, T. Orimoto, A. Tishelman-charny, T. Wamorkar, B. Wang, A. Wisecarver, D. Wood, S. Bhattacharya, J. Bueghly, O. Charaf, T. Gunter, K. A. Hahn, N. Odell, M. H. Schmitt, K. Sung, M. Trovato, M. Velasco, R. Bucci, N. Dev, R. Goldouzian, M. Hildreth, K. Hurtado Anampa, C. Jessop, D. J. Karmgard, K. Lannon, W. Li, N. Loukas, N. Marinelli, F. Meng, C. Mueller, Y. Musienko, M. Planer, R. Ruchti, P. Siddireddy, G. Smith, S. Taroni, M. Wayne, A. Wightman, M. Wolf, A. Woodard, J. Alimena, L. Antonelli, B. Bylsma, L. S. Durkin, S. Flowers, B. Francis, C. Hill, W. Ji, T. Y. Ling, W. Luo, B. L. Winer, S. Cooperstein, G. Dezoort, P. Elmer, J. Hardenbrook, N. Haubrich, S. Higginbotham, A. Kalogeropoulos, S. Kwan, D. Lange, M. T. Lucchini, J. Luo, D. Marlow, K. Mei, I. Ojalvo, J. Olsen, C. Palmer, P. Piroué, J. Salfeld-Nebgen, D. Stickland, C. Tully, S. Malik, S. Norberg, A. Barker, V. E. Barnes, S. Das, L. Gutay, M. Jones, A. W. Jung, A. Khatiwada, B. Mahakud, D. H. Miller, N. Neumeister, C. C. Peng, S. Piperov, H. Qiu, J. F. Schulte, J. Sun, F. Wang, R. Xiao, W. Xie, T. Cheng, J. Dolen, N. Parashar, Z. Chen, K. M. Ecklund, S. Freed, F. J. M. Geurts, M. Kilpatrick, A. Kumar, W. Li, B. P. Padley, R. Redjimi, J. Roberts, J. Rorie, W. Shi, Z. Tu, A. Zhang, A. Bodek, P. de Barbaro, R. Demina, Y. t. Duh, J. L. Dulemba, C. Fallon, T. Ferbel, M. Galanti, A. Garcia-Bellido, J. Han, O. Hindrichs, A. Khukhunaishvili, E. Ranken, P. Tan, R. Taus, B. Chiarito, J. P. Chou, Y. Gershtein, E. Halkiadakis, A. Hart, M. Heindl, E. Hughes, S. Kaplan, R. Kunnawalkam Elayavalli, S. Kyriacou, I. Laflotte, A. Lath, R. Montalvo, K. Nash, M. Osherson, H. Saka, S. Salur, S. Schnetzer, D. Sheffield, S. Somalwar, R. Stone, S. Thomas, P. Thomassen, H. Acharya, A. G. Delannoy, J. Heideman, G. Riley, S. Spanier, O. Bouhali, A. Celik, M. Dalchenko, M. De Mattia, A. Delgado, S. Dildick, R. Eusebi, J. Gilmore, T. Huang, T. Kamon, S. Luo, D. Marley, R. Mueller, D. Overton, L. Perniè, D. Rathjens, A. Safonov, N. Akchurin, J. Damgov, F. De Guio, P. R. Dudero, S. Kunori, K. Lamichhane, S. W. Lee, T. Mengke, S. Muthumuni, T. Peltola, S. Undleeb, I. Volobouev, Z. Wang, A. Whitbeck, S. Greene, A. Gurrola, R. Janjam, W. Johns, C. Maguire, A. Melo, H. Ni, K. Padeken, F. Romeo, P. Sheldon, S. Tuo, J. Velkovska, M. Verweij, Q. Xu, M. W. Arenton, P. Barria, B. Cox, R. Hirosky, M. Joyce, A. Ledovskoy, H. Li, C. Neu, T. Sinthuprasith, Y. Wang, E. Wolfe, F. Xia, R. Harr, P. E. Karchin, N. Poudyal, J. Sturdy, P. Thapa, S. Zaleski, J. Buchanan, C. Caillol, D. Carlsmith, S. Dasu, I. De Bruyn, L. Dodd, B. Gomber, M. Grothe, M. Herndon, A. Hervé, U. Hussain, P. Klabbers, A. Lanaro, K. Long, R. Loveless, T. Ruggles, A. Savin, V. Sharma, N. Smith, W. H. Smith, N. Woods, CMS Collaboration

**Affiliations:** 1 Yerevan Physics Institute, Yerevan, Armenia; 2 Institut für Hochenergiephysik, Vienna, Austria; 3 Institute for Nuclear Problems, Minsk, Belarus; 4 Universiteit Antwerpen, Antwerp, Belgium; 5 Vrije Universiteit Brussel, Brussels, Belgium; 6 Université Libre de Bruxelles, Brussels, Belgium; 7 Ghent University, Ghent, Belgium; 8 Université Catholique de Louvain, Louvain-la-Neuve, Belgium; 9 Centro Brasileiro de Pesquisas Fisicas, Rio de Janeiro, Brazil; 10 Universidade do Estado do Rio de Janeiro, Rio de Janeiro, Brazil; 11 Universidade Estadual Paulista, Universidade Federal do ABC, São Paulo, Brazil; 12 Institute for Nuclear Research and Nuclear Energy, Bulgarian Academy of Sciences, Sofia, Bulgaria; 13 University of Sofia, Sofia, Bulgaria; 14 Beihang University, Beijing, China; 15 Institute of High Energy Physics, Beijing, China; 16 State Key Laboratory of Nuclear Physics and Technology, Peking University, Beijing, China; 17 Tsinghua University, Beijing, China; 18 Universidad de Los Andes, Bogotá, Colombia; 19 Faculty of Electrical Engineering, Mechanical Engineering and Naval Architecture, University of Split, Split, Croatia; 20 Faculty of Science, University of Split, Split, Croatia; 21 Institute Rudjer Boskovic, Zagreb, Croatia; 22 University of Cyprus, Nicosia, Cyprus; 23 Charles University, Prague, Czech Republic; 24 Escuela Politecnica Nacional, Quito, Ecuador; 25 Universidad San Francisco de Quito, Quito, Ecuador; 26 Academy of Scientific Research and Technology of the Arab Republic of Egypt, Egyptian Network of High Energy Physics, Cairo, Egypt; 27 National Institute of Chemical Physics and Biophysics, Tallinn, Estonia; 28 Department of Physics, University of Helsinki, Helsinki, Finland; 29 Helsinki Institute of Physics, Helsinki, Finland; 30 Lappeenranta University of Technology, Lappeenranta, Finland; 31 IRFU, CEA, Université Paris-Saclay, Gif-sur-Yvette, France; 32 Laboratoire Leprince-Ringuet, Ecole polytechnique, CNRS/IN2P3, Université Paris-Saclay, Palaiseau, France; 33 Université de Strasbourg, CNRS, IPHC UMR 7178, Strasbourg, France; 34 Centre de Calcul de l’Institut National de Physique Nucleaire et de Physique des Particules, CNRS/IN2P3, Villeurbanne, France; 35 Université de Lyon, Université Claude Bernard Lyon 1, CNRS-IN2P3, Institut de Physique Nucléaire de Lyon, Villeurbanne, France; 36 Georgian Technical University, Tbilisi, Georgia; 37 Tbilisi State University, Tbilisi, Georgia; 38 I. Physikalisches Institut, RWTH Aachen University, Aachen, Germany; 39 III. Physikalisches Institut A, RWTH Aachen University, Aachen, Germany; 40 III. Physikalisches Institut B, RWTH Aachen University, Aachen, Germany; 41 Deutsches Elektronen-Synchrotron, Hamburg, Germany; 42 University of Hamburg, Hamburg, Germany; 43 Karlsruher Institut fuer Technologie, Karlsruhe, Germany; 44 Institute of Nuclear and Particle Physics (INPP), NCSR Demokritos, Aghia Paraskevi, Greece; 45 National and Kapodistrian University of Athens, Athens, Greece; 46 National Technical University of Athens, Athens, Greece; 47 University of Ioánnina, Ioánnina, Greece; 48 MTA-ELTE Lendület CMS Particle and Nuclear Physics Group, Eötvös Loránd University, Budapest, Hungary; 49 Wigner Research Centre for Physics, Budapest, Hungary; 50 Institute of Nuclear Research ATOMKI, Debrecen, Hungary; 51 Institute of Physics, University of Debrecen, Debrecen, Hungary; 52 Indian Institute of Science (IISc), Bangalore, India; 53 National Institute of Science Education and Research, HBNI, Bhubaneswar, India; 54 Panjab University, Chandigarh, India; 55 University of Delhi, Delhi, India; 56 Saha Institute of Nuclear Physics, HBNI, Kolkata, India; 57 Indian Institute of Technology Madras, Madras, India; 58 Bhabha Atomic Research Centre, Mumbai, India; 59 Tata Institute of Fundamental Research-A, Mumbai, India; 60 Tata Institute of Fundamental Research-B, Mumbai, India; 61 Indian Institute of Science Education and Research (IISER), Pune, India; 62 Institute for Research in Fundamental Sciences (IPM), Tehran, Iran; 63 University College Dublin, Dublin, Ireland; 64 INFN Sezione di Bari, Università di Bari, Politecnico di Bari, Bari, Italy; 65 INFN Sezione di Bologna, Università di Bologna, Bologna, Italy; 66 INFN Sezione di Catania, Università di Catania, Catania, Italy; 67 INFN Sezione di Firenze, Università di Firenze, Florence, Italy; 68 INFN Laboratori Nazionali di Frascati, Frascati, Italy; 69 INFN Sezione di Genova, Università di Genova, Genoa, Italy; 70 INFN Sezione di Milano-Bicocca, Università di Milano-Bicocca, Milan, Italy; 71 INFN Sezione di Napoli, Università di Napoli ’Federico II’ , Naples, Italy, Università della Basilicata, Potenza, Italy, Università G. Marconi, Rome, Italy; 72 INFN Sezione di Padova, Università di Padova, Padova, Italy, Università di Trento, Trento, Italy; 73 INFN Sezione di Pavia, Università di Pavia, Pavia, Italy; 74 INFN Sezione di Perugia, Università di Perugia, Perugia, Italy; 75 INFN Sezione di Pisa, Università di Pisa, Scuola Normale Superiore di Pisa, Pisa, Italy; 76 INFN Sezione di Roma, Sapienza Università di Roma, Rome, Italy; 77 INFN Sezione di Torino, Università di Torino, Torino, Italy, Università del Piemonte Orientale, Novara, Italy; 78 INFN Sezione di Trieste, Università di Trieste, Trieste, Italy; 79 Kyungpook National University, Daegu, Korea; 80 Institute for Universe and Elementary Particles, Chonnam National University, Kwangju, Korea; 81 Hanyang University, Seoul, Korea; 82 Korea University, Seoul, Korea; 83 Sejong University, Seoul, Korea; 84 Seoul National University, Seoul, Korea; 85 University of Seoul, Seoul, Korea; 86 Sungkyunkwan University, Suwon, Korea; 87 Riga Technical University, Riga, Latvia; 88 Vilnius University, Vilnius, Lithuania; 89 National Centre for Particle Physics, Universiti Malaya, Kuala Lumpur, Malaysia; 90 Universidad de Sonora (UNISON), Hermosillo, Mexico; 91 Centro de Investigacion y de Estudios Avanzados del IPN, Mexico City, Mexico; 92 Universidad Iberoamericana, Mexico City, Mexico; 93 Benemerita Universidad Autonoma de Puebla, Puebla, Mexico; 94 Universidad Autónoma de San Luis Potosí, San Luis Potosí, Mexico; 95 University of Auckland, Auckland, New Zealand; 96 University of Canterbury, Christchurch, New Zealand; 97 National Centre for Physics, Quaid-I-Azam University, Islamabad, Pakistan; 98 National Centre for Nuclear Research, Swierk, Poland; 99 Institute of Experimental Physics, Faculty of Physics, University of Warsaw, Warsaw, Poland; 100 Laboratório de Instrumentação e Física Experimental de Partículas, Lisbon, Portugal; 101 Joint Institute for Nuclear Research, Dubna, Russia; 102 Petersburg Nuclear Physics Institute, Gatchina (St. Petersburg), Russia; 103 Institute for Nuclear Research, Moscow, Russia; 104 Institute for Theoretical and Experimental Physics, Moscow, Russia; 105 Moscow Institute of Physics and Technology, Moscow, Russia; 106 National Research Nuclear University ’Moscow Engineering Physics Institute’ (MEPhI), Moscow, Russia; 107 P.N. Lebedev Physical Institute, Moscow, Russia; 108 Skobeltsyn Institute of Nuclear Physics, Lomonosov Moscow State University, Moscow, Russia; 109 Novosibirsk State University (NSU), Novosibirsk, Russia; 110 Institute for High Energy Physics of National Research Centre ’Kurchatov Institute’, Protvino, Russia; 111 National Research Tomsk Polytechnic University, Tomsk, Russia; 112 Faculty of Physics and VINCA Institute of Nuclear Sciences, University of Belgrade, Belgrade, Serbia; 113 Centro de Investigaciones Energéticas Medioambientales y Tecnológicas (CIEMAT), Madrid, Spain; 114 Universidad Autónoma de Madrid, Madrid, Spain; 115 Universidad de Oviedo, Oviedo, Spain; 116 Instituto de Física de Cantabria (IFCA), CSIC-Universidad de Cantabria, Santander, Spain; 117 Department of Physics, University of Ruhuna, Matara, Sri Lanka; 118 CERN, European Organization for Nuclear Research, Geneva, Switzerland; 119 Paul Scherrer Institut, Villigen, Switzerland; 120 ETH Zurich-Institute for Particle Physics and Astrophysics (IPA), Zurich, Switzerland; 121 Universität Zürich, Zurich, Switzerland; 122 National Central University, Chung-Li, Taiwan; 123 National Taiwan University (NTU), Taipei, Taiwan; 124 Department of Physics, Faculty of Science, Chulalongkorn University, Bangkok, Thailand; 125 Physics Department, Science and Art Faculty, Çukurova University, Adana, Turkey; 126 Physics Department, Middle East Technical University, Ankara, Turkey; 127 Bogazici University, Istanbul, Turkey; 128 Istanbul Technical University, Istanbul, Turkey; 129 Institute for Scintillation Materials of National Academy of Science of Ukraine, Kharkov, Ukraine; 130 National Scientific Center, Kharkov Institute of Physics and Technology, Kharkov, Ukraine; 131 University of Bristol, Bristol, United Kingdom; 132 Rutherford Appleton Laboratory, Didcot, United Kingdom; 133 Imperial College, London, United Kingdom; 134 Brunel University, Uxbridge, United Kingdom; 135 Baylor University, Waco, USA; 136 Catholic University of America, Washington DC, USA; 137 The University of Alabama, Tuscaloosa, USA; 138 Boston University, Boston, USA; 139 Brown University, Providence, USA; 140 University of California, Davis, Davis, USA; 141 University of California, Los Angeles, USA; 142 University of California, Riverside, Riverside, USA; 143 University of California, San Diego, La Jolla, USA; 144 Department of Physics, University of California, Santa Barbara, Santa Barbara, USA; 145 California Institute of Technology, Pasadena, USA; 146 Carnegie Mellon University, Pittsburgh, USA; 147 University of Colorado Boulder, Boulder, USA; 148 Cornell University, Ithaca, USA; 149 Fermi National Accelerator Laboratory, Batavia, USA; 150 University of Florida, Gainesville, USA; 151 Florida International University, Miami, USA; 152 Florida State University, Tallahassee, USA; 153 Florida Institute of Technology, Melbourne, USA; 154 University of Illinois at Chicago (UIC), Chicago, USA; 155 The University of Iowa, Iowa City, USA; 156 Johns Hopkins University, Baltimore, USA; 157 The University of Kansas, Lawrence, USA; 158 Kansas State University, Manhattan, USA; 159 Lawrence Livermore National Laboratory, Livermore, USA; 160 University of Maryland, College Park, USA; 161 Massachusetts Institute of Technology, Cambridge, USA; 162 University of Minnesota, Minneapolis, USA; 163 University of Mississippi, Oxford, USA; 164 University of Nebraska-Lincoln, Lincoln, USA; 165 State University of New York at Buffalo, Buffalo, USA; 166 Northeastern University, Boston, USA; 167 Northwestern University, Evanston, USA; 168 University of Notre Dame, Notre Dame, USA; 169 The Ohio State University, Columbus, USA; 170 Princeton University, Princeton, USA; 171 University of Puerto Rico, Mayagüez, USA; 172 Purdue University, West Lafayette, USA; 173 Purdue University Northwest, Hammond, USA; 174 Rice University, Houston, USA; 175 University of Rochester, Rochester, USA; 176 Rutgers, The State University of New Jersey, Piscataway, USA; 177 University of Tennessee, Knoxville, USA; 178 Texas A&M University, College Station, USA; 179 Texas Tech University, Lubbock, USA; 180 Vanderbilt University, Nashville, USA; 181 University of Virginia, Charlottesville, USA; 182 Wayne State University, Detroit, USA; 183 University of Wisconsin-Madison, Madison, WI USA; 184 CERN, 1211 Geneva 23, Switzerland

## Abstract

A search for new physics in top quark production is performed in proton-proton collisions at $13\,\text {TeV}$. The data set corresponds to an integrated luminosity of $35.9{\,\text {fb}^{-1}}$ collected in 2016 with the CMS detector. Events with two opposite-sign isolated leptons (electrons or muons), and $\mathrm{b}$ quark jets in the final state are selected. The search is sensitive to new physics in top quark pair production and in single top quark production in association with a $\mathrm{W}$ boson. No significant deviation from the standard model expectation is observed. Results are interpreted in the framework of effective field theory and constraints on the relevant effective couplings are set, one at a time, using a dedicated multivariate analysis. This analysis differs from previous searches for new physics in the top quark sector by explicitly separating $\mathrm{t}\mathrm{W}$ from $\mathrm{t}{\bar{\mathrm{t}}}$ events and exploiting the specific sensitivity of the $\mathrm{t}\mathrm{W}$ process to new physics.

## Introduction

Because of its large mass, close to the electroweak (EW) symmetry breaking scale, the top quark is predicted to play an important role in several new physics scenarios. If the new physics scale is in the available energy range of the CERN LHC, the existence of new physics could be directly observed via the production of new particles. Otherwise, new physics could affect standard model (SM) interactions indirectly, through modifications of SM couplings or enhancements of rare SM processes. In this case, it is useful to introduce a model independent approach to parametrize and constrain possible deviations from SM predictions, independently of the fundamental theory of new physics.

Several searches for new physics in the top quark sector including new non-SM couplings of the top quark have been performed at the Tevatron and LHC colliders [1–4, 4–10]. Most of the previous analyses followed the anomalous coupling approach in which the SM Lagrangian is extended for possible new interactions. Another powerful framework to parametrize deviations with respect to the SM prediction is the effective field theory (EFT) [11, 12]. Constraints obtained on anomalous couplings can be translated to the effective coupling bounds [1, 13]. Several groups have performed global fits of top quark EFT to unfolded experimental data from the Tevatron and LHC colliders [14, 15]. Due to the limited access to data and details of the associated uncertainties, correlations between various cross section measurements and related uncertainties are neglected in a global fit on various unfolded measurements. On the other hand, EFT operators could affect backgrounds for some processes constructively or destructively while cross sections are measured with the SM assumptions for background processes. Inside the CMS Collaboration and with direct access to data, all mentioned points could be considered properly.

**Fig. 1:**
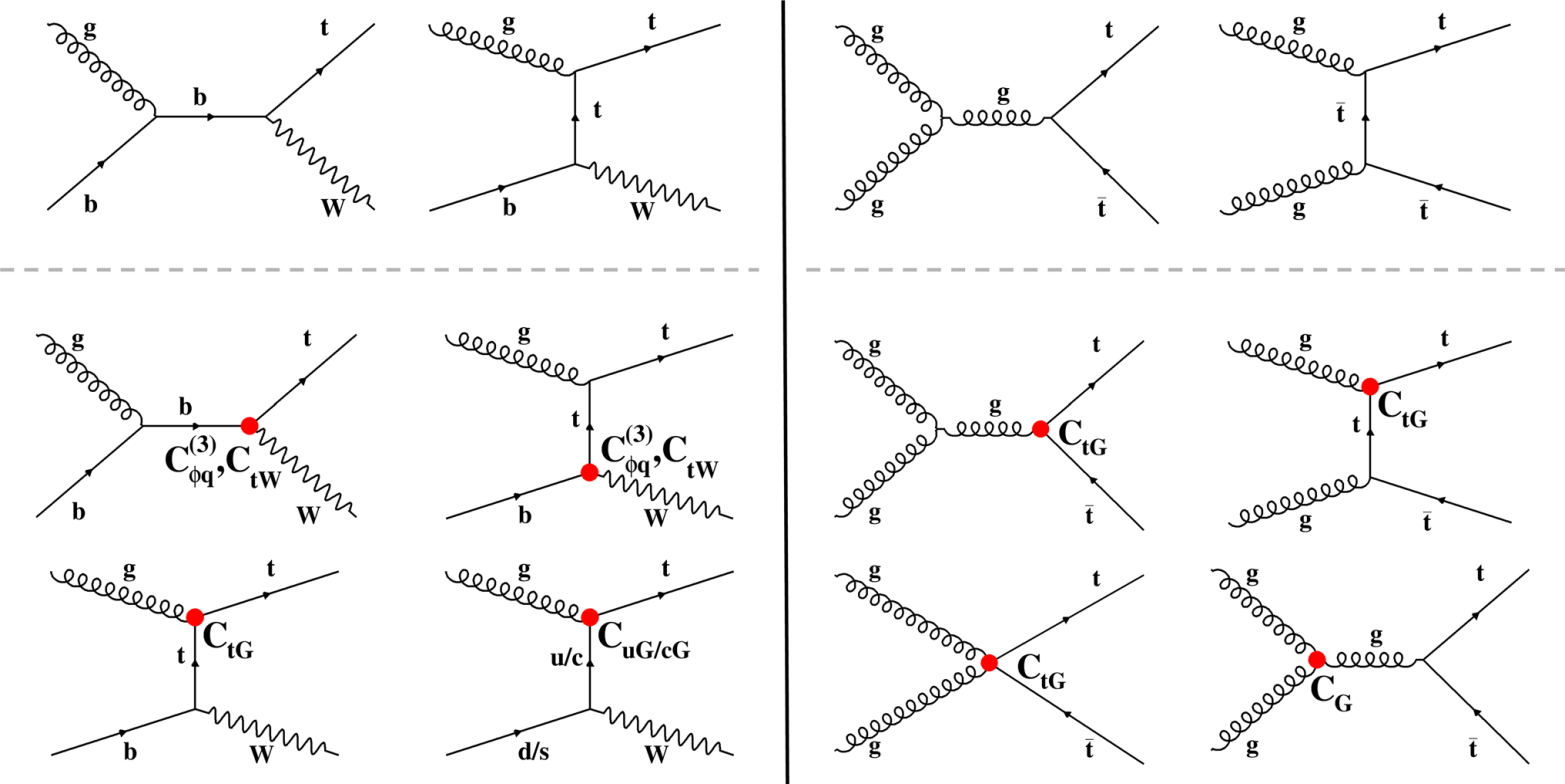
Representative Feynman diagrams for the $\mathrm{t}\mathrm{W}$ (left panel) and $\mathrm{t}{\bar{\mathrm{t}}}$ (right panel) production at leading order. The upper row presents the SM diagrams, the middle and lower rows present diagrams corresponding to the $O_{\phi \mathrm{q}}^{(3)}$, $O_{\mathrm{t}\mathrm{W}}$,$O_{\mathrm{t}\mathrm{G}}$, $O_{\mathrm{G}}$ and $O_{\mathrm {u/cG}}$ contributions

In this paper, the EFT approach is followed to search for new physics in the top quark sector in the dilepton final states. In Refs. [13, 16], all dimension-six operators that contribute to top quark pair ($\mathrm{t}{\bar{\mathrm{t}}}$) production and single top quark production in association with a $\mathrm{W}$ boson ($\mathrm{t}\mathrm{W}$) are investigated. The operators and the related effective Lagrangians, which are relevant for dilepton final states, can be written as [12]:

1 $$\begin{aligned} O_{\phi \mathrm{q}}^{(3)}&= (\phi ^+\uptau ^iD_\upmu \phi )({\bar{\mathrm{q}}}\upgamma ^\upmu \uptau ^i\mathrm{q}), \nonumber \\ L_{\mathrm {eff}}&=\frac{{\mathrm {C}}_{\phi \mathrm{q}}^{(3)}}{{\sqrt{2}}{\varLambda }^2} \mathrm{g}v^2{\bar{\mathrm{b}}}\upgamma ^\upmu P_{\mathrm {L}}\mathrm{t}\mathrm{W}^-_\upmu +\text {h.c.}, \end{aligned}$$

2 $$\begin{aligned} O_{\mathrm{t}\mathrm{W}}&= ({\bar{\mathrm{q}}}\sigma ^{\upmu \upnu }\uptau ^i\mathrm{t})\tilde{\phi }\mathrm{W}^i_{\upmu \upnu }, \nonumber \\ L_{\mathrm {eff}}&=-2\frac{{\mathrm {C}}_{\mathrm{t}\mathrm{W}}}{{\varLambda }^2}v{\bar{\mathrm{b}}}\sigma ^{\upmu \upnu }P_{\mathrm {R}}\mathrm{t}\partial _\upnu \mathrm{W}^-_\upmu +\text {h.c.}, \end{aligned}$$

3 $$\begin{aligned} O_{\mathrm{t}\mathrm{G}}&= ({\bar{\mathrm{q}}}\sigma ^{\upmu \upnu }\lambda ^a \mathrm{t})\tilde{\phi }\mathrm{G}^a_{\upmu \upnu }, \nonumber \\ L_{\mathrm {eff}}&= \frac{{\mathrm {C}}_{\mathrm{t}\mathrm{G}}}{{\sqrt{2}}{\varLambda }^2}v\left( {\bar{\mathrm{t}}}\sigma ^{\upmu \upnu }\lambda ^a \mathrm{t}\right) \mathrm{G}_{\upmu \upnu }^a+\text {h.c.}, \end{aligned}$$

4 $$\begin{aligned} O_{\mathrm{G}}&= f_{abc} \mathrm{G}^{a\upnu }_{\upmu } \mathrm{G}^{b\uprho }_{\upnu } \mathrm{G}^{\mathrm{c}\upmu }_{\uprho }, \nonumber \\ L_{\mathrm {eff}}&= \frac{{\mathrm {C}}_{\mathrm{G}}}{{\varLambda }^2} f_{abc}\mathrm{G}^{a\upnu }_\upmu \mathrm{G}^{b\uprho }_\upnu \mathrm{G}^{\mathrm{c}\upmu }_{\uprho }, \end{aligned}$$

5 $$\begin{aligned} O_{\mathrm{u}(\mathrm{c})\mathrm{G}}&= ({\bar{\mathrm{q}}}\sigma ^{\upmu \upnu }\lambda ^a \mathrm{t})\tilde{\phi }\mathrm{G}^a_{\upmu \upnu }, \nonumber \\ L_{\mathrm {eff}}&= \frac{{\mathrm {C}}_{\mathrm{u}(\mathrm{c})\mathrm{G}}}{{\sqrt{2}}{\varLambda }^2}v\left( {\bar{\mathrm{u}}}\left( {\bar{\mathrm{c}}}\right) \sigma ^{\upmu \upnu }\lambda ^a \mathrm{t}\right) \mathrm{G}_{\upmu \upnu }^a+\text {h.c.}, \end{aligned}$$

where $D_\upmu =\partial _\upmu -i\mathrm{g}_s\frac{1}{2}\lambda ^a\mathrm{G}_\upmu ^a-i\mathrm{g}\frac{1}{2}\uptau ^i\mathrm{W}_\upmu ^i-i\mathrm{g}^{'}YB_\upmu$, $\mathrm{W}_{\upmu \upnu }^i=\partial _\upmu \mathrm{W}_\upnu ^i-\partial _\upnu \mathrm{W}^i_\upmu +\mathrm{g}\epsilon _{ijk}\mathrm{W}^j_\upmu \mathrm{W}^k_\upnu$, $\mathrm{G}_{\upmu \upnu }^a=\partial _\upmu \mathrm{G}_\upnu ^a-\partial _\upnu \mathrm{G}^a_\upmu +\mathrm{g}_sf^{abc}\mathrm{G}^b_\upmu \mathrm{G}^c_\upnu$, $\sigma ^{\upmu \upnu } = \frac{1}{2}[\upgamma ^{\upmu },\upgamma ^{\upnu }]$, $P_{\mathrm {L,R}}=\frac{1}{2} (1\mp \upgamma ^5)$, and the symbols $\mathrm{q}$, $\mathrm{t}$ and $\phi$ ($\tilde{\phi }=\epsilon \phi ^*$) in the operators represent the left-handed quark doublet, the right-handed top quark singlet, and the Higgs boson doublet fields, respectively. The parameters ${\hbox {C}}_{\phi \mathrm{q}}^{(3)}$, ${\hbox {C}}_{\mathrm{t}\mathrm{W}}$, ${\hbox {C}}_{\mathrm{t}\mathrm{G}}$, ${\hbox {C}}_{\mathrm{G}}$ and ${\hbox {C}}_{\mathrm{u}(\mathrm{c})\mathrm{G}}$ stand for the dimensionless Wilson coefficients, also called effective couplings. The variable ${\varLambda }$ represents the energy scale beyond which new physics becomes relevant. A detailed description of the operators is given in Refs. [13, 16, 17]. In this analysis, four-fermion operators involved in $\mathrm{t}{\bar{\mathrm{t}}}$ production are not probed. Up to order ${\varLambda }^{-2}$, the $\mathrm{t}\mathrm{W}$ and $\mathrm{t}{\bar{\mathrm{t}}}$ production cross sections and most of the differential observables considered in this analysis do not receive CP-odd contributions. Therefore, we only probe CP-even operators with real coefficients. The operators $O_{\phi \mathrm{q}}^{(3)}$ and $O_{\mathrm{t}\mathrm{W}}$ modify the SM interaction between the $\mathrm{W}$ boson, top quark, and $\mathrm{b}$ quark ($\mathrm{W}\mathrm{t}\mathrm{b}$). We consider the EFT effects in the production of top quarks not in their decays [18]. The operator $O_{\mathrm{t}\mathrm{G}}$ is called the chromomagnetic dipole moment operator of the top quark and can arise from various models of new physics [19, 20]. The triple-gluon field strength operator $O_{\mathrm{G}}$ represents the only genuinely gluonic CP conserving term that can appear at dimension six within an effective strong interaction Lagrangian. Although it is shown that jet production at the LHC can set a tight constraint on the ${\hbox {C}}_{\mathrm{G}}$ [21], $\mathrm{t}{\bar{\mathrm{t}}}$ production is also considered as a promising channel [22, 23]. The operators $O_{\mathrm{u}\mathrm{G}}$ and $O_{\mathrm{c}\mathrm{G}}$ lead to flavor-changing neutral current (FCNC) interactions of the top quark and contribute to $\mathrm{t}\mathrm{W}$ production. The effect of introducing new couplings ${\hbox {C}}_{\phi \mathrm{q}}^{(3)}$, ${\hbox {C}}_{\mathrm{t}\mathrm{W}}$, ${\hbox {C}}_{\mathrm{t}\mathrm{G}}$ and ${\hbox {C}}_{\mathrm{u}(\mathrm{c})\mathrm{G}}$ can be investigated in $\mathrm{t}\mathrm{W}$ production. The chromomagnetic dipole moment operator of the top quark also affects $\mathrm{t}{\bar{\mathrm{t}}}$ production. In the case of ${\hbox {C}}_{\mathrm{G}}$ coupling, only $\mathrm{t}{\bar{\mathrm{t}}}$ production is modified. It should be noted that the $O_{\mathrm{t}\mathrm{W}}$ and $O_{\mathrm{t}\mathrm{G}}$ operators with imaginary coefficients lead to CP-violating effects. Representative Feynman diagrams for SM and new physics contributions in $\mathrm{t}\mathrm{W}$ and $\mathrm{t}{\bar{\mathrm{t}}}$ production are shown in Fig. 1.

A variety of limits have been set on the $\mathrm{W}\mathrm{t}\mathrm{b}$ anomalous coupling through single top quark *t*-channel production and measurements of the $\mathrm{W}$ boson polarization from top quark decay by the D0 [1], ATLAS [2, 3] and CMS [4, 5] Collaborations. Direct limits on the top quark chromomagnetic dipole moment have been obtained by the CMS Collaboration at 7 and $13\,\text {TeV}$ using top quark pair production events [6, 10]. Searches for top quark FCNC interactions have been performed at the Tevatron [7, 8] and LHC [4, 9] via single top quark production and limits are set on related anomalous couplings.

In this paper, a search for new physics in top quark production using an EFT framework is reported. This is the first such search for new physics that uses the $\mathrm{t}\mathrm{W}$ process. Final states with two opposite-sign isolated leptons (electrons or muons) in association with jets identified as originating from the fragmentation of a bottom quark (“$\mathrm{b}$jets”) are analyzed. The search is sensitive to new physics contributions to $\mathrm{t}\mathrm{W}$ and $\mathrm{t}{\bar{\mathrm{t}}}$ production, and the six effective couplings, ${\hbox {C}}_{\mathrm{G}}$, ${\hbox {C}}_{\phi \mathrm{q}}^{(3)}$, ${\hbox {C}}_{\mathrm{t}\mathrm{W}}$, ${\hbox {C}}_{\mathrm{t}\mathrm{G}}$, ${\hbox {C}}_{\mathrm{u}\mathrm{G}}$, and ${\hbox {C}}_{\mathrm{c}\mathrm{G}}$, are constrained assuming one non-zero effective coupling at a time. The effective couplings affect both the rate of $\mathrm{t}{\bar{\mathrm{t}}}$ and $\mathrm{t}\mathrm{W}$ production and the kinematic distributions of final state particles. For the ${\hbox {C}}_{\phi \mathrm{q}}^{(3)}$, ${\hbox {C}}_{\mathrm{t}\mathrm{W}}$, ${\hbox {C}}_{\mathrm{t}\mathrm{G}}$, and ${\hbox {C}}_{\mathrm{G}}$ effective couplings, the deviation from the SM prediction is dominated by the interference term between SM and new physics diagrams, which is linear with respect to the effective coupling. Therefore, the kinematic distributions of the final-state particles vary as a function of the Wilson coefficients. For small effective couplings the kinematic distributions approach those predicted by the SM. On the other hand, the new physics terms due to the ${\hbox {C}}_{\mathrm{u}\mathrm{G}}$ and ${\hbox {C}}_{\mathrm{c}\mathrm{G}}$ effective couplings do not interfere with the SM $\mathrm{t}\mathrm{W}$ process, and the kinematic distributions of final-state particles are determined by the new physics terms independently of the SM prediction. In this analysis, we use the rates of $\mathrm{t}\mathrm{W}$ and $\mathrm{t}{\bar{\mathrm{t}}}$ production to probe the ${\hbox {C}}_{\phi \mathrm{q}}^{(3)}$, ${\hbox {C}}_{\mathrm{t}\mathrm{W}}$, ${\hbox {C}}_{\mathrm{t}\mathrm{G}}$, and ${\hbox {C}}_{\mathrm{G}}$ effective couplings. Variations in both rate and kinematic distributions of final-state particles are employed to probe the ${\hbox {C}}_{\mathrm{u}\mathrm{G}}$ and ${\hbox {C}}_{\mathrm{c}\mathrm{G}}$ effective couplings. The analysis utilizes proton-proton ($\mathrm{p}\mathrm{p}$) collision data collected by the CMS experiment in 2016 at a center-of-mass energy of $13\,\text {TeV}$, corresponding to an integrated luminosity of $35.9{\,\text {fb}^{-1}}$.

The paper is structured as follows. In Sect. 2, a description of the CMS detector is given and the simulated samples used in the analysis are detailed. The event selection and the SM background estimation are presented in Sect. 3. Section 4 presents a description of the signal extraction procedure. An overview of the systematic uncertainty treatment is given in Sect. 5. Finally, the constraints on the effective couplings are presented in Sect. 6, and a summary is given in Sect. 7.

## The CMS detector and event simulation

The central feature of the CMS apparatus is a superconducting solenoid of 6 m internal diameter, providing a magnetic field of 3.8 T. Within the solenoid volume are a silicon pixel and strip tracker, a lead tungstate crystal electromagnetic calorimeter (ECAL), and a brass and scintillator hadron calorimeter, each composed of a barrel and two endcap sections. Forward calorimeters extend the pseudorapidity ($\eta$) coverage provided by the barrel and endcap detectors. Muons are detected in gas-ionisation chambers embedded in the steel flux-return yoke outside the solenoid. A more detailed description of the CMS detector, together with a definition of the coordinate system used and the relevant kinematic variables, can be found in Ref. [24].

The Monte Carlo (MC) samples for the $\mathrm{t}{\bar{\mathrm{t}}}$, $\mathrm{t}\mathrm{W}$ and diboson ($\mathrm {VV} = \mathrm{W}\mathrm{W}$, $\mathrm{W}\mathrm{Z}$, $\mathrm{Z}\mathrm{Z}$) SM processes are simulated using the Powheg-Box event generator (v1 for $\mathrm{t}\mathrm{W}$, v2 for $\mathrm{t}{\bar{\mathrm{t}}}$ and diboson) [25–28] at the next-to-leading order (NLO), interfaced with pythia (v8.205) [29] to simulate parton showering and to match soft radiations with the contributions from the matrix elements. The pythia tune CUETP8M1 [30] is used for all samples except for the $\mathrm{t}{\bar{\mathrm{t}}}$ sample, for which the tune CUETP8M2 [31] is used. The NNPDF3.0 [32] set of the parton distribution functions (PDFs) is used. The $\mathrm{t}{\bar{\mathrm{t}}}$ and $\mathrm{t}\mathrm{W}$ samples are normalized to the next-to-next-to-leading order (NNLO) and approximate NNLO cross section calculations, respectively [33, 34]. In order to better describe the transverse momentum ($p_{\mathrm {T}}$) distribution of the top quark in $\mathrm{t}{\bar{\mathrm{t}}}$ events, the top quark $p_{\mathrm {T}}$ spectrum simulated with powheg is reweighted to match the differential top quark $p_{\mathrm {T}}$ distribution at NNLO quantum chromoDynamics (QCD) accuracy and including EW corrections calculated in Ref. [35]. Other SM background contributions, from Drell–Yan (DY), $\mathrm{t}{\bar{\mathrm{t}}}{+}\hbox {V}$, $\mathrm{t}{\bar{\mathrm{t}}}{+}\upgamma$, and $\mathrm{W}+\upgamma$ processes, are simulated at NLO using the MadGraph 5_amc@nlo (v2.2.2) event generator [36–38], interfaced with pythia v8 for parton showering and hadronization. The events include the effects of additional $\mathrm{p}\mathrm{p}$ interactions in the same or nearby bunch crossings (pileup) and are weighted according to the observed pileup distribution in the analyzed data. The CMS detector response is simulated using Geant4  (v9.4) [39, 40], followed by a detailed trigger simulation. Simulated events are reconstructed with the same algorithms as used for data.

In order to calculate the total cross sections for the $\mathrm{t}{\bar{\mathrm{t}}}$ and $\mathrm{t}\mathrm{W}$ processes and generate events in the presence of new effective interactions, the operators of Eqs. 1–5 have been implemented in the universal FeynRules output (UFO) format [41] through the FeynRules package [42]. The output EFT model is used in the MadGraph 5_amc@nlo (v2.2.2) event generator [36, 37]. If we allow for the presence of one operator at a time, the total cross section up to $\mathcal {O}({\varLambda }^{-4})$ can be parametrized as

6 $$\begin{aligned} \sigma =\sigma _{\mathrm {SM}}+ {\mathrm {C}}_{i}\sigma _i^{(1)}+ {\mathrm {C}}_{i}^2\sigma _{i}^{(2)}, \end{aligned}$$

where the ${\hbox {C}}_i$s are effective couplings introduced in Eqs. 1–5. Here, $\sigma _i^{(1)}$ is the contribution to the cross section due to the interference term between the SM diagrams and diagrams with one EFT vertex. The cross section $\sigma _i^{(2)}$ is the pure new physics contribution. We use the most precise available SM cross section prediction, which are $\sigma _{\text {SM}}^{\mathrm{t}{\bar{\mathrm{t}}}}=832^{+20}_{-29}\,\text {(scales)}\pm 35\,(\text {PDF}+\alpha _S) \, \hbox {pb}$ and $\sigma _{\text {SM}}^{\mathrm{t}\mathrm{W}}=71.7\pm 1.8\,\text {(scales)}\pm 3.4\,(\text {PDF}+\alpha _S) \, \hbox {pb}$ for $\mathrm{t}{\bar{\mathrm{t}}}$ and $\mathrm{t}\mathrm{W}$ production, respectively [33, 34], where the $\alpha _S$ is strong coupling constant. The first uncertainty reflects the uncertainties in the factorization and renormalization scales. In the framework of EFT, the $\sigma _i^{(1)}$ and $\sigma _i^{(2)}$ terms have been calculated at NLO accuracy for all of the operators, except $O_{\mathrm{G}}$ [16, 43, 44]. At the time the work for this paper was concluded, there was no available UFO model including the $O_{\mathrm{G}}$ operator at the NLO. The values of $\sigma _i^{(1)}$ and $\sigma _i^{(2)}$ for various effective couplings at LO and available *K* factors are given in Table 1.

**Table 1:** Contribution to the cross section due to the interference between the SM diagrams and diagrams with one EFT vertex ($\sigma _i^{(1)}$), and the pure new physics ($\sigma _i^{(2)}$) for $\mathrm{t}{\bar{\mathrm{t}}}$ and $\mathrm{t}\mathrm{W}$ production [in pb] for the various effective couplings for ${\varLambda }= 1\,\text {TeV}$. The respective *K* factors ($\sigma _i^{\mathrm {NLO}}/\sigma _i^{\mathrm {LO}}$) are also shown

Channel	Contribution	$C_{\mathrm{G}}$	$C_{\phi \mathrm{q}}^{(3)}$	$C_{\mathrm{t}\mathrm{W}}$	$C_{\mathrm{t}\mathrm{G}}$	$C_{\mathrm{u}\mathrm{G}}$	$C_{\mathrm{c}\mathrm{G}}$
$\mathrm{t}{\bar{\mathrm{t}}}$	$\sigma _i^{(1) \mathrm { -LO}}$	31.9 pb	—	—	137 pb	—	—
	$K^{(1)}$	—	—	—	1.48	—	—
	$\sigma _i^{(2)\mathrm { -LO}}$	102.3 pb	—	—	16.4 pb	—	—
	$K^{(2)}$	—	—	—	1.44	—	—
$\mathrm{t}\,\mathrm{W}$	$\sigma _i^{(1) \mathrm { -LO}}$	—	6.7 pb	−4.5 pb	3.3 pb	0	0
	$K^{(1)}$	—	1.32	1.27	1.27	0	0
	$\sigma _i^{(2) \mathrm { -LO}}$	—	0.2 pb	1 pb	1.2 pb	16.2 pb	4.6 pb
	$K^{(2)}$	—	1.31	1.18	1.06	1.27	1.27

## Event selection and background estimation

The event selection for this analysis is similar to the one used in Ref. [10]. The events of interest are recorded by the CMS detector using a combination of dilepton and single-lepton triggers. Single-lepton triggers require at least one isolated electron (muon) with $p_{\mathrm {T}} >27\,(24)\,\text {GeV}$. The dilepton triggers select events with at least two leptons with loose isolation requirements and $p_{\mathrm {T}}$ for the leading and sub-leading leptons greater than 23 and 12 (17 and 8) $\text {GeV}$ for the $\mathrm{e}\mathrm{e}$ ($\upmu \upmu$) final state. In the $\mathrm{e}\upmu$ final state, in the case of the leading lepton being an electron, the events are triggered if the electron-muon pair has a $p_{\mathrm {T}}$ greater than 23 and $8 \,\text {GeV}$ for the electron and muon, respectively. In the case of the leading lepton being a muon, the trigger thresholds are 23 and $12\,\text {GeV}$ for the muon and electron, respectively [45].

Offline, the particle-flow (PF) algorithm [46] aims to reconstruct and identify each individual particle with an optimized combination of information from the various elements of the CMS detector. Electron candidates are reconstructed using tracking and ECAL information [47]. Requirements on electron identification variables based on shower shape and track-cluster matching are further applied to the reconstructed electron candidates, together with isolation criteria [10, 47]. Electron candidates are selected with $p_{\mathrm {T}} >20\,\text {GeV}$ and $|\eta |< 2.4$. Electron candidates within the range $1.444< |\eta |< 1.566$, which corresponds to the transition region between the barrel and endcap regions of the ECAL, are not considered. Information from the tracker and the muon spectrometer are combined in a global fit to reconstruct muon candidates [48]. Muon candidates are further required to have a high-quality fit including a minimum number of hits in both systems, and to be isolated [10, 48]. The muons used in this analysis are selected inside the fiducial region of the muon spectrometer, $|\eta |<2.4$, with a minimum $p_{\mathrm {T}}$ of $20\,\text {GeV}$.

The PF candidates are clustered into jets using the anti-$k_{\mathrm {T}}$ algorithm with a distance parameter of 0.4 [49–51]. Jets are calibrated in data and simulation, accounting for energy deposits of particles from pileup [52]. Jets with $p_{\mathrm {T}} >30\,\text {GeV}$ and $|\eta | <2.4$ are selected; loose jets are defined as jets with the $p_{\mathrm {T}}$ range between 20 and $30\,\text {GeV}$. Jets originating from the hadronization of $\mathrm{b}$ quarks are identified using the combined secondary vertex algorithm [53]; this algorithm combines information from track impact parameters and secondary vertices identified within a given jet. The chosen working point provides a signal identification efficiency of approximately 68% with a probability to misidentify light-flavor jets as $\mathrm{b}$jets of approximately 1% [53]. The missing transverse momentum vector ${\vec {p}}_{\mathrm {T}}^{\text {miss}}$ is defined as the projection on the plane perpendicular to the proton beams axis of the negative vector sum of the momenta of all reconstructed PF candidates in the event [54]. Corrections to the jet energies are propagated to ${\vec {p}}_{\mathrm {T}}^{\text {miss}}$. Its magnitude is referred to as $p_{\mathrm {T}} ^\text {miss}$.

Events are required to have at least two leptons (electrons or muons) with opposite sign and with an invariant mass above $20\,\text {GeV}$. The leading lepton must fulfill $p_{\mathrm {T}} >25\,\text {GeV}$. For the same-flavor lepton channels, to suppress the DY background, the dilepton invariant mass must not be within $15\,\text {GeV}$ of the $\mathrm{Z}$ boson mass and a minimal value (of $60\,\text {GeV}$) on $p_{\mathrm {T}} ^\text {miss}$ is applied.

**Fig. 2:**
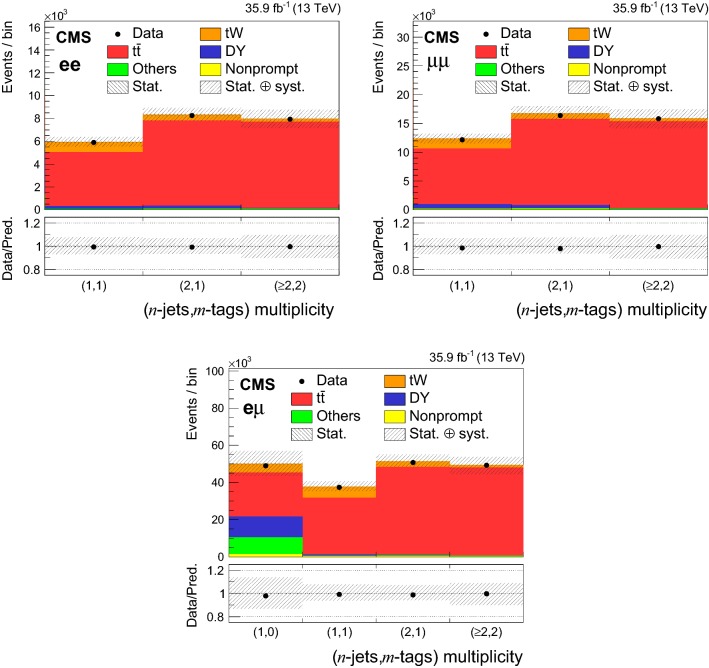
The observed number of events and SM background predictions in the search regions of the analysis for the $\mathrm{e}\mathrm{e}$ (upper left), $\upmu \upmu$ (upper right), and $\mathrm{e}\upmu$ (lower) channels. The hatched bands correspond to the quadratic sum of statistical and systematic uncertainties in the event yield for the SM background predictions. The ratios of data to the sum of the predicted yields are shown at the bottom of each plot. The narrow hatched bands represent the contribution from the statistical uncertainty in the MC simulation

The events are divided into the $\mathrm{e}\mathrm{e}$, $\mathrm{e}\upmu$, and $\upmu \upmu$ channels according to the flavors of the two leptons with the highest $p_{\mathrm {T}}$ and are further categorized in different bins depending on the number of jets (“*n*-jets”) and number $\mathrm{b}$-tagged jets (“*m*-tags”) in the final state. The largest number of $\mathrm{t}\mathrm{W}$ events is expected in the category with exactly one $\mathrm{b}$-tagged jet (1-jet,1-tag) followed by the category with two jets, of which one a $\mathrm{b}$jet (2-jets,1-tag). Events in the categories with more than two jets and exactly two $\mathrm{b}$-tagged jets are dominated by the $\mathrm{t}{\bar{\mathrm{t}}}$ process ($\ge$2-jets,2-tags). Categories with zero $\mathrm{b}$jets are dominated by DY events in the $\mathrm{e}\mathrm{e}$ and $\upmu \upmu$ channels and are not used in the analysis. However, in the $\mathrm{e}\upmu$ channel, the contamination of DY events is lower and a significant number of $\mathrm{t}\mathrm{W}$ events is present in the category with one jet and zero $\mathrm{b}$-tagged jets (1-jet,0-tag). The latter category is included in this analysis. In Fig. 2, the data in the ten search regions are shown together with the SM background predictions.

The contributions of SM processes leading to two prompt leptons in the final state are estimated from simulated samples and are normalised to the integrated luminosity of the data. These contributions originate mainly from $\mathrm{t}{\bar{\mathrm{t}}}$, $\mathrm{t}\mathrm{W}$ and DY production. Other SM processes, such as diboson, $\mathrm{t}{\bar{\mathrm{t}}}{+} \hbox {V}$ and $\mathrm{t}{\bar{\mathrm{t}}}{+}\upgamma$ have significantly smaller contributions.

To correct the DY simulation for the efficiency of the $p_{\mathrm {T}} ^\text {miss}$ threshold and for the mismodeling of the heavy-flavour content, scale factors are derived using the ratio of the numbers of simulated events inside and outside the dilepton invariant mass window, 76–$106 \,\text {GeV}$. The observed event yield inside the window is scaled to estimate the DY background outside the mass window [55].

The nonprompt lepton backgrounds which contain fake lepton(s) from a misreconstructed $\upgamma$ or jet(s) are also considered. The contribution of misidentified or converted $\upgamma$ events from the $\mathrm{W}\upgamma$ process is estimated from MC simulation. The contribution from $\mathrm{W}$+jets and multijet processes is estimated by a data-based technique using events with same-sign leptons. The method is based on the assumption that the probability of assigning positive or negative charge to the fake lepton is equal. Therefore, the background contribution from fake leptons in the final selection (opposite-sign sample) can be estimated from the corresponding sample with same-sign leptons. In this latter same-sign event sample, the remaining small contribution from prompt-lepton backgrounds is subtracted from data using MC samples.

After all selections, the expected numbers of events from $\mathrm{t}\mathrm{W}$, $\mathrm{t}{\bar{\mathrm{t}}}$, DY, and remaining background contributions mentioned above, as well as the total number of background events are reported in Table 2 for the $\mathrm{e}\mathrm{e}$, $\mathrm{e}\upmu$, and $\upmu \upmu$ channels and for the various (*n*-jets,*m*-tags) categories. We find generally very good agreement between data and predictions, within the uncertainties of the data.

**Table 2:** Number of expected events from $\mathrm{t}\mathrm{W}$, $\mathrm{t}{\bar{\mathrm{t}}}$ and DY production, from the remaining backgrounds (other), total background contribution and observed events in data after all selections for the $\mathrm{e}\mathrm{e}$, $\mathrm{e}\upmu$, and $\upmu \upmu$ channels and for different (*n*-jets,*m*-tags) categories. The uncertainties correspond to the statistical contribution only for the individual background predictions and to the quadratic sum of the statistical and systematic contributions for the total background predictions

Channel	(*n*-jets,*m*-tags)	Prediction					Data
		$\mathrm{t}\,\mathrm{W}$	$\mathrm{t}{\bar{\mathrm{t}}}$	DY	Other	Total yield	
$\mathrm{e}\mathrm{e}$	(1,1)	884±8	4741 ± 15	258 ± 50	53 ± 5	5936 ± 470	5902
	(2,1)	518 ± 6	7479 ± 19	241 ± 53	94 ± 5	8331 ± 597	8266
	($\ge$2,2)	267 ± 4	7561 ± 18	46 ± 24	99 ± 4	7973 ± 819	7945
$\mathrm{e}\upmu$	(1,0)	4835 ± 20	23557 ± 35	11352 ± 277	10294 ± 72	50038 ± 6931	48973
	(1,1)	6048 ± 22	30436 ± 38	561 ± 66	629 ± 13	37673 ± 2984	37370
	(2,1)	3117 ± 16	47206 ± 48	278 ± 48	781 ± 9	51382 ± 3714	50725
	($\ge$2,2)	1450 ± 10	47310 ± 46	32 ± 22	598 ± 9	49391 ± 5010	49262
$\upmu \upmu$	(1,1)	1738 ± 12	9700 ± 21	744 ± 90	183 ± 5	12366 ± 879	12178
	(2,1)	989 ± 9	14987 ± 27	501 ± 75	275 ± 5	16751 ± 1276	16395
	($\ge$2,2)	508 ± 6	15136 ± 26	82 ± 24	163 ± 5	15889 ± 1714	15838

## Signal extraction using neural networks tools

The purpose of the analysis is to search for deviations from the SM predictions in the $\mathrm{t}\mathrm{W}$ and $\mathrm{t}{\bar{\mathrm{t}}}$ production due to new physics, parametrized with the presence of new effective couplings. In order to investigate the effect of the non-zero effective couplings, it is important to find suitable variables with high discrimination power between the signal and the background. Depending on the couplings, the total yield or the distribution of the output of a neural network (NN) algorithm is employed, as summarized in Table 3. The NN algorithm used in this analysis is a multilayer perceptron [56].

**Table 3:** Summary of the observables used to probe the effective couplings in various (*n*-jets,*m*-tags) categories in the $\mathrm{e}\mathrm{e}$, $\mathrm{e}\upmu$, and $\upmu \upmu$ channels

Eff. coupling	Channel	Categories				
		1-jet, 0-tag	1-jet, 1-tag	2-jets, 1-tag	>2-jets, 1-tag	$\ge$2-jets, 2-tags
${\hbox {C}}_{\mathrm{G}}$	$\mathrm{e}\mathrm{e}$	—	Yield	Yield	—	Yield
	$\mathrm{e}\upmu$	Yield	Yield	Yield	—	Yield
	$\upmu \upmu$	—	Yield	Yield	—	Yield
${\hbox {C}}_{\phi \mathrm{q}}^{(3)}$, ${\hbox {C}}_{\mathrm{t}\mathrm{W}}$, ${\hbox {C}}_{\mathrm{t}\mathrm{G}}$	$\mathrm{e}\mathrm{e}$	—	${\hbox {NN}}_{11}$	${\hbox {NN}}_{21}$	—	Yield
	$\mathrm{e}\upmu$	${\hbox {NN}}_{10}$	${\hbox {NN}}_{11}$	${\hbox {NN}}_{21}$	—	Yield
	$\upmu \upmu$	—	${\hbox {NN}}_{11}$	${\hbox {NN}}_{21}$	—	Yield
${\hbox {C}}_{\mathrm{u}\mathrm{G}}$, ${\hbox {C}}_{\mathrm{c}\mathrm{G}}$	$\mathrm{e}\mathrm{e}$	—	${\hbox {NN}}_{\text {FCNC}}$			—
	$\mathrm{e}\upmu$	—	${\hbox {NN}}_{\text {FCNC}}$			—
	$\upmu \upmu$	—	${\hbox {NN}}_{\text {FCNC}}$			—

All the effective couplings introduced in Sect. 1 can contribute to $\mathrm{t}\mathrm{W}$ production except the triple gluon field strength operator, $O_{\mathrm{G}}$ which only affects $\mathrm{t}{\bar{\mathrm{t}}}$ production. As observed in previous analysis [22] and confirmed here, the top quark $p_{\mathrm {T}}$ distribution is sensitive to the triple-gluon field-strength operator. The kinematic distributions of final-state particles show less discrimination power than the top quark $p_{\mathrm {T}}$ distribution. In addition, they vary with the value of ${\hbox {C}}_{\mathrm{G}}$ and approach the SM prediction for decreasing values of ${\hbox {C}}_{\mathrm{G}}$. Therefore, we use the total yield in various categories to constrain the ${\hbox {C}}_{\mathrm{G}}$ effective coupling.

**Fig. 3:**
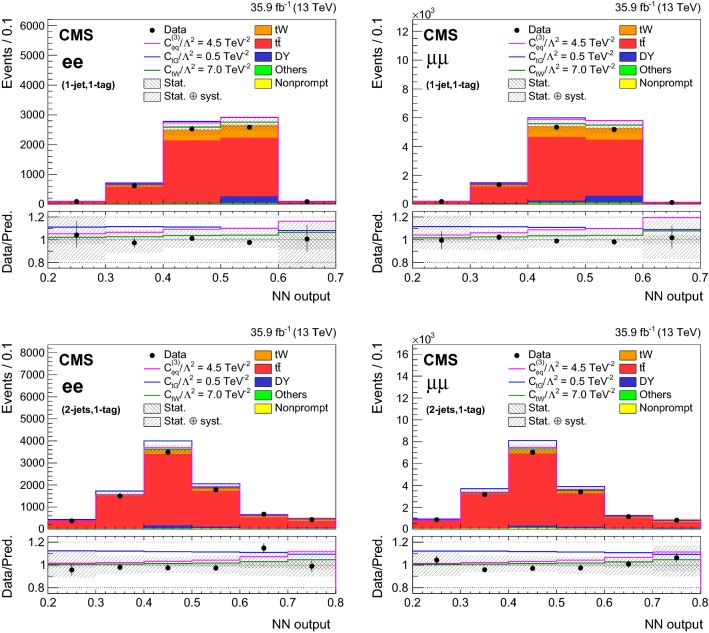
The NN output distributions for data and simulation for the $\mathrm{e}\mathrm{e}$ (left) and $\upmu \upmu$ (right) channels in 1-jet, 1-tag (upper) and 2-jets, 1-tag (lower) categories. The hatched bands correspond to the quadratic sum of the statistical and systematic uncertainties in the event yield for the sum of signal and background predictions. The ratios of data to the sum of the predicted yields are shown at the lower panel of each graph. The narrow hatched bands represent the contribution from the statistical uncertainty in the MC simulation. In each plot, the expected distributions assuming specific values for the effective couplings (given in the legend) are shown as the solid curves

**Fig. 4:**
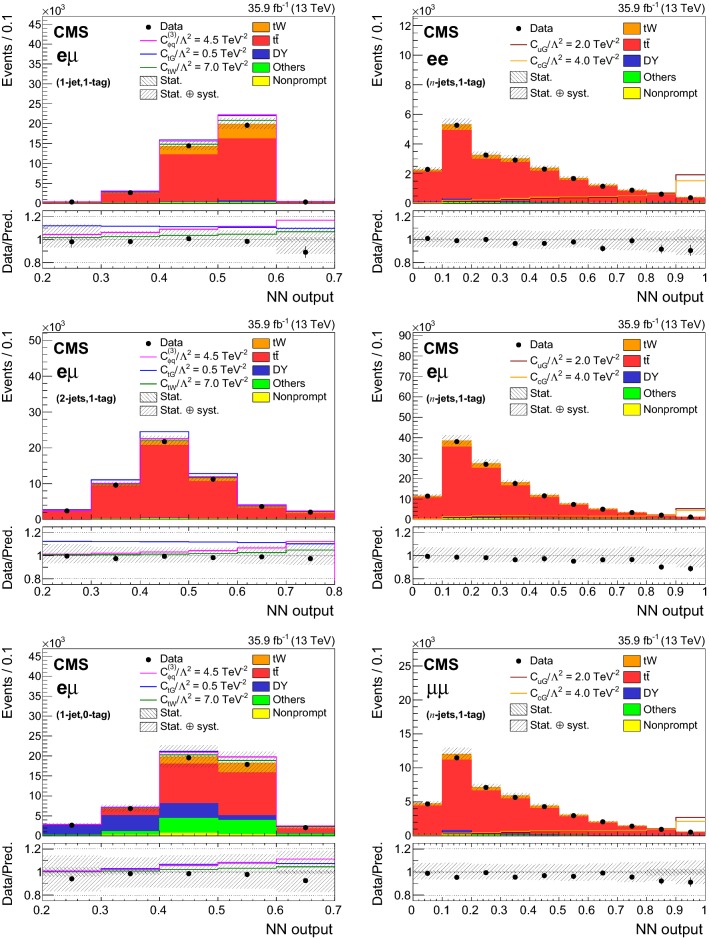
The NN output distributions for (left) data and simulation for the $\mathrm{e}\upmu$ channel in 1-jet, 1-tag (upper) and 2-jets, 1-tag (middle) and 1-jet, 0-tag (lower) categories; and for (right) data, simulation, and FCNC signals in the *n*-jets, 1-tag category used in the limit setting for the $\mathrm{e}\mathrm{e}$ (upper), $\mathrm{e}\upmu$ (middle), and $\upmu \upmu$ (lower) channels. The hatched bands correspond to the quadratic sum of the statistical and systematic uncertainties in the event yield for the sum of signal and background predictions. The ratios of data to the sum of the predicted yields are shown at the lower panel of each graph. The narrow hatched bands represent the contribution from the statistical uncertainty in the MC simulation. In each plot, the expected distributions assuming specific values for the effective couplings (given in the legend) are shown as the solid curves

The deviation from the SM $\mathrm{t}\mathrm{W}$ production because of the interference between the SM and the $O_{\mathrm{t}\mathrm{G}}$, $O_{\phi \mathrm{q}}^{(3)}$, and $O_{\mathrm{t}\mathrm{W}}$ operators is of the order of $1/{\varLambda }^2$. It is assumed that the new physics scale ${\varLambda }$ is larger than the scale we probe. Therefore, $1/{\varLambda }^4$ contributions from the new physics terms are small compared to the contribution from the interference term. The operator $O_{\phi \mathrm{q}}^{(3)}$ is similar to the SM $\mathrm{W}\mathrm{t}\mathrm{b}$ operator and leads to a rescaling of the SM $\mathrm{W}\mathrm{t}\mathrm{b}$ vertex [13]. The $O_{\mathrm{t}\mathrm{W}}$ and $O_{\mathrm{t}\mathrm{G}}$ operators lead to the right-handed Wtb interaction and a tensor-like ttg interaction, respectively, which are absent in the SM at the first order. Their effects have been investigated via the various kinematic distributions of the final-state particles considered in this analysis and are found to be not distinguishable from the SM $\mathrm{t}\mathrm{W}$ and $\mathrm{t}{\bar{\mathrm{t}}}$ processes for unconstrained values of the effective couplings within the current precision on data. After the selection described in Section 3, the dominant background comes from $\mathrm{t}{\bar{\mathrm{t}}}$ production, with a contribution of about 90%. In order to observe deviations from SM $\mathrm{t}\mathrm{W}$ production in the presence of the $O_{\phi \mathrm{q}}^{(3)}$, $O_{\mathrm{t}\mathrm{W}}$, and $O_{\mathrm{t}\mathrm{G}}$ effective operators, we need to separate $\mathrm{t}\mathrm{W}$ events from the large number of $\mathrm{t}{\bar{\mathrm{t}}}$ events. Two independent NNs are trained to separate $\mathrm{t}{\bar{\mathrm{t}}}$ events (the background) and $\mathrm{t}\mathrm{W}$ events (considered as the signal) in the (1-jet, 1-tag) (${\hbox {NN}}_{11}$) and (2-jets, 1-tag) (${\hbox {NN}}_{21}$) categories, which have significant signal contributions [57]. For the $\mathrm{e}\upmu$ channel, another NN is used for the (1-jet, 0-tag) (${\hbox {NN}}_{10}$) category, in which the $\mathrm{t}{\bar{\mathrm{t}}}$, $\mathrm{W}\mathrm{W}$, and DY events are combined and are considered as the background. A comparison between the observed data and the SM background prediction of the NN output shape in various (*n*-jets, *m*-tags) categories is shown for the $\mathrm{e}\mathrm{e}$ and $\upmu \upmu$ channels in Fig. 3 and for the $\mathrm{e}\upmu$ channel in Fig. 4 (left column).

The presence of the $O_{\mathrm{u}\mathrm{G}}$ and $O_{\mathrm{c}\mathrm{G}}$ operators changes the initial-state particle (see Fig. 1), and leads to different kinematic distributions for the final-state particles, compared to the SM $\mathrm{t}\mathrm{W}$ process. For these FCNC operators, new physics effects on final-state particle distributions are expected to be distinguishable from SM processes. In order to search for new physics due to the $O_{\mathrm{u}\mathrm{G}}$ and $O_{\mathrm{c}\mathrm{G}}$ effective operators, an NN (${\hbox {NN}}_{\mathrm {FCNC}}$) is used to separate SM backgrounds ($\mathrm{t}{\bar{\mathrm{t}}}$ and $\mathrm{t}\mathrm{W}$ events together) and new physics signals for events with exactly one $\mathrm{b}$-tagged jet with no requirement on the number of light-flavor jets (*n*-jets, 1-tag). The comparison of the NN output for data, SM background and signal ($\mathrm{t}\mathrm{W}$ events via FCNC interactions) is shown in Fig. 4 (right column) for the $\mathrm{e}\mathrm{e}$, $\mathrm{e}\upmu$, and $\upmu \upmu$ channels.

The various input variables for training the NN introduced above are described below and are shown in Table  4.

- M$_{\ell \ell }$ (where $\ell = \mathrm{e}$  or $\upmu$), invariant mass of dilepton system;
- $p_{\mathrm {T}} ^{\ell \ell }$, $p_{\mathrm {T}}$ of dilepton system;
- $${\varDelta }p_{\mathrm {T}} (\ell _1,\, \ell _2)$$, $$p_{\mathrm {T}} ^{\text {leading lepton}} - p_{\mathrm {T}} ^{\text {sub-leading lepton}}$$;
- $p_{\mathrm {T}} ^{\ell _1}$, $p_{\mathrm {T}}$ of leading lepton;
- Centrality($\ell _1$, jet$_1$), scalar sum of $p_{\mathrm {T}}$ of the leading lepton and leading jet, over total energy of selected leptons and jets;
- Centrality($\ell _1,\, \ell _2$), scalar sum of $p_{\mathrm {T}}$ of the leading and sub-leading leptons, over total energy of selected leptons and jets;
- ${\varDelta }{\varPhi }(\ell \ell$, jet$_1$), ${\varDelta }{\varPhi }$ between dilepton system and leading jet where ${\varPhi }$ is azimuthal angle;
- $p_{\mathrm {T}}$ ($\ell \ell$, jet$_1$), $p_{\mathrm {T}}$ of dilepton and leading jet system;
- $p_{\mathrm {T}}$ ($\ell _1$, jet$_1$), $p_{\mathrm {T}}$ of leading lepton and leading jet system;
- Centrality($\ell \ell$, jet$_1$), scalar sum of $p_{\mathrm {T}}$ of the dilepton system and leading jet, over total energy of selected leptons and jets;
- $${\varDelta }$$R($$\ell _1,\, \ell _2$$), $$\sqrt{\smash [b]{ \, ( \eta ^{\ell _1} - \eta ^{\ell _2})^2 \, + \, ({\varPhi }^{\ell _1} - {\varPhi }^{\ell _2})^2}}$$;
- $${\varDelta }$$R($$\ell _1$$, jet$$_1$$), $$\sqrt{\smash [b]{ \, ( \eta ^{\ell _1} - \eta ^{\text {jet}_1})^2 \, + \, ({\varPhi }^{\ell _1} - {\varPhi }^{ \text {jet}_1})^2}}$$;
- M($\ell _1$, jet$_1$), invariant mass of leading lepton and leading jet;
- M(jet$_1$, jet$_2$), invariant mass of leading jet and sub-leading jet;
- $${\varDelta }$$R($$\ell _1$$, jet$$_2$$), $$\sqrt{\smash [b]{ \, ( \eta ^{\ell _1} - \eta ^{\text {jet}_2})^2 \, + \, ({\varPhi }^{\ell _1} - {\varPhi }^{\text {jet}_2})^2}}$$;
- $${\varDelta }$$R($$\ell \ell $$, jet$$_1$$), $$\sqrt{\smash [b]{ \, ( \eta ^{\ell \ell } - \eta ^{\text {jet}_1})^2 \, + \, ({\varPhi }^{\ell \ell } - {\varPhi }^{\text {jet}_1})^2}}$$;
- $${\varDelta }p_{\mathrm {T}} (\ell _2,~ \text {jet}_2)$$, $$p_{\mathrm {T}} ^{\ell _2} - p_{\mathrm {T}} ^{\text {jet}_2}$$;
- M($\ell _2$, jet$_1$), invariant mass of sub-leading lepton and leading jet.

**Table 4:** Input variables for the NN used in the analysis in various bins of *n*-jets and *m*-tags. The symbols “$\times$” indicate the input variables used in the four NNs

Variable	${\hbox {NN}}_{10}$	${\hbox {NN}}_{11}$	${\hbox {NN}}_{21}$	${\hbox {NN}}_{\text {FCNC}}$
M$_{\ell \ell }$	$\times$			$\times$
$p_{\mathrm {T}} ^{\ell \ell }$	$\times$		$\times$	$\times$
${\varDelta }p_{\mathrm {T}} (\ell _1,\, \ell _2)$	$\times$			$\times$
$p_{\mathrm {T}} ^{\ell _1}$	$\times$		$\times$	$\times$
Centrality($\ell _1$, jet$_1$)	$\times$			$\times$
Centrality($\ell _1,\, \ell _2$)	$\times$			$\times$
${\varDelta }{\varPhi }(\ell \ell$, jet$_1$)	$\times$	$\times$	$\times$	
$p_{\mathrm {T}}$ ($\ell \ell$, jet$_1$)		$\times$		$\times$
$p_{\mathrm {T}}$ ($\ell _1$, jet$_1$)		$\times$		
Centrality($\ell \ell$, jet$_1$)		$\times$		
${\varDelta }$R($\ell _1,\, \ell _2$)		$\times$		
${\varDelta }$R($\ell _1$, jet$_1$)		$\times$		
M($\ell _1$, jet$_1$)			$\times$	
M(jet$_1$, jet$_2$)			$\times$	
${\varDelta }$R($\ell _1$, jet$_2$)			$\times$	
${\varDelta }$R($\ell \ell$, jet$_1$)			$\times$	$\times$
${\varDelta }p_{\mathrm {T}} (\ell _2$, jet$_2$)			$\times$	
M($\ell _2$, jet$_1$)				$\times$

## Systematic uncertainties

The normalization and shape of the signal and the backgrounds are both affected by different sources of systematic uncertainty. For each source, an induced variation can be parametrized, and treated as a nuisance parameter in the fit that is described in the next section.

A systematic uncertainty of 2.5% is assigned to the integrated luminosity and is used for signal and background rates [58]. The efficiency corrections for trigger and offline selection of leptons were estimated by comparing the efficiency measured in data and in MC simulation using $\mathrm{Z}\rightarrow \ell \ell$ events, based on a “tag-and-probe” method as in Ref. [59]. The scale factors obtained are varied by one standard deviation to take into account the corresponding uncertainties in the efficiency. The jet energy scale and resolution uncertainties depend on $p_{\mathrm {T}}$ and $\eta$ of the jet and are computed by shifting the energy of each jet and propagating the variation to ${\vec {p}}_{\mathrm {T}}^{\text {miss}}$ coherently [60].

The uncertainty in the $\mathrm{b}$ tagging is estimated by varying the $\mathrm{b}$ tagging scale factors within one standard deviation [53]. Effects of the uncertainty in the distribution of the number of pileup interactions are evaluated by varying the effective inelastic $\mathrm{p}\mathrm{p}$ cross section used to predict the number of pileup interactions in MC simulation by ± 4.6% of its nominal value [61].

The uncertainty in the DY contribution in categories with one or two $\mathrm{b}$-tagged jets is considered to be 50 and 30% in the $\mathrm{e}\upmu$ and same-flavor dilepton channels, respectively [10, 57]. For the DY normalization in the (1-jet, 0-tag) category, an uncertainty of 15% is assigned [62]. In addition, systematic uncertainties related to the PDF, and to the renormalization and factorization scale uncertainty are taken into account for DY process in the (1-jet, 0-tag) category. The uncertainty in the yield of nonprompt lepton backgrounds is considered to be 50% [57]. Contributions to the background from $\mathrm{t}{\bar{\mathrm{t}}}$ production in association with a boson, as well as diboson production, are estimated from simulation and a systematic uncertainty of 50% is conservatively assigned [63].

Various uncertainties originate from the theoretical predictions. The effect of the renormalization and factorization scale uncertainty from the $\mathrm{t}{\bar{\mathrm{t}}}$ and $\mathrm{t}\mathrm{W}$ MC generators is estimated by varying the scales used during the generation of the simulation sample independently by a factor 0.5, 1 or 2. Unphysical cases, where one scale fluctuates up while the other fluctuates down, are not considered. The top quark $p_{\mathrm {T}}$ reweighting procedure, discussed in Sect. 2, is applied on top of the nominal powheg prediction at NLO to account for the higher-order corrections.

The uncertainty in the PDFs for each simulated signal process is obtained using the replicas of the NNPDF 3.0 set [64]. The most recent measurement of the top quark mass by CMS yields a total uncertainty of $\pm 0.49 \,\text {GeV}$ [65]. We consider variations of the top quark mass due to this uncertainty and they are found to be insignificant. At NLO QCD, $\mathrm{t}\mathrm{W}$ production is expected to interfere with $\mathrm{t}{\bar{\mathrm{t}}}$ production [66]. Two schemes for defining the $\mathrm{t}\mathrm{W}$ signal in a way that distinguishes it from the $\mathrm{t}{\bar{\mathrm{t}}}$ production have been compared in the analysis: the “diagram removal” (DR), in which all doubly resonant NLO $\mathrm{t}\mathrm{W}$ diagrams are removed, and the “diagram subtraction” (DS), where a gauge-invariant subtractive term modifies the NLO $\mathrm{t}\mathrm{W}$ cross section to locally cancel the contribution from $\mathrm{t}{\bar{\mathrm{t}}}$ production [66–68]. The DR method is used for the nominal $\mathrm{t}\mathrm{W}$ sample and the difference with respect to the sample simulated using the DS method is taken as a systematic uncertainty. The model parameter $h_{\text {damp}}$ in $\mathrm{t}{\bar{\mathrm{t}}}$ powheg [25] that controls the matching of the matrix elements to the pythia parton showers is varied from a top quark mass default value of $172.5 \,\text {GeV}$ by factors of 0.5 and 2 for estimating the uncertainties from the matching between jets from matrix element calculations and parton shower emissions. The renormalization scale for QCD emissions in the initial- and final-state radiation (ISR and FSR) is varied up and down by factors of 2 and $\sqrt{2}$, respectively, to account for parton shower QCD scale variation error in both $\mathrm{t}{\bar{\mathrm{t}}}$ and $\mathrm{t}\mathrm{W}$ samples [69]. In addition, several dedicated $\mathrm{t}{\bar{\mathrm{t}}}$ samples are used to estimate shower modeling uncertainties in both underlying event and color re-connections [10, 31, 69]. To estimate model uncertainties, $\mathrm{t}\mathrm{W}$ and $\mathrm{t}{\bar{\mathrm{t}}}$ samples are generated with powheg as described in Sect. 2, varying the relevant model parameters with respect to the nominal samples.

## Constraints on the effective couplings

The six Wilson coefficients sensitive to new physics contributions in top quark interactions, as defined in Eqs. 1–5, are tested separately in the observed data. The event yields and the NN output distributions in each analysis category, summarized in Table 3, are used to construct a binned likelihood function. All sources of systematic uncertainty, described in Sect. 5, are taken into account as nuisance parameters in the fit. A simultaneous binned maximum-likelihood fit is performed to find the best fit value for each Wilson coefficient together with 68 and 95% confidence intervals (CIs) [70]. In this section, distributions of the log-likelihood functions are shown with one nonzero effective coupling at a time for ${\varLambda }= 1\,\text {TeV}$.

**Fig. 5:**
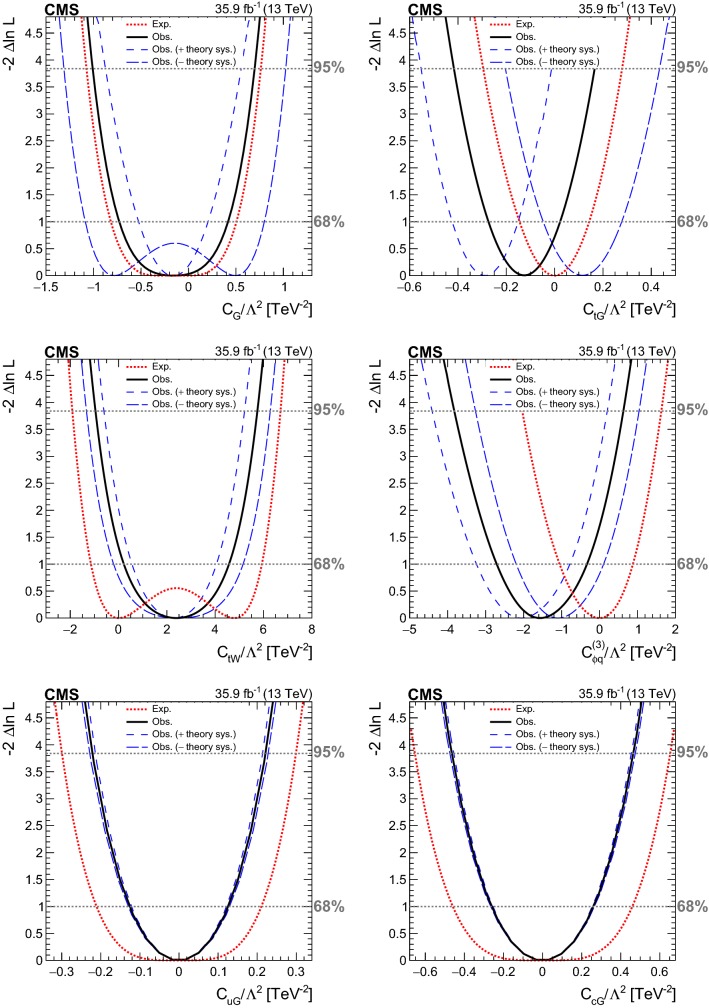
Observed (solid) and expected (dotted) log likelihoods for the effective couplings: ${\hbox {C}}_{\mathrm{G}}$ (upper left), ${\hbox {C}}_{\mathrm{t}\mathrm{G}}$ (upper right), ${\hbox {C}}_{\mathrm{t}\mathrm{W}}$ (middle left), ${\hbox {C}}_{\phi \mathrm{q}}$ (middle right), ${\hbox {C}}_{\mathrm{u}\mathrm{G}}$ (lower left), and ${\hbox {C}}_{\mathrm{c}\mathrm{G}}$ (lower right). The dashed curves represent fits to the observed data with the variations of normalization due to the theoretical uncertainties

The SM cross section prediction for the $\mathrm{t}{\bar{\mathrm{t}}}$ and $\mathrm{t}\mathrm{W}$ processes, $\sigma _i^{(1)}$ and $\sigma _i^{(2)}$ (see Table 1), are accompanied by uncertainties in scales and PDFs. These theoretical uncertainties can affect the bounds on the Wilson coefficients. In order to study this effect, the fit is performed on data, while theoretical uncertainties are varied within one standard deviation and are shown together with the nominal results in the likelihood scan plots in Fig. 5. The nominal theoretical cross sections for $\mathrm{t}{\bar{\mathrm{t}}}$ and $\mathrm{t}\mathrm{W}$ processes are varied by [$+4.8\%,-5.5\%$] and [$+5.4\%,-5.4\%$] , respectively. These variations cover the uncertainties arising from the variations of factorization and renormalization scales and PDFs [10, 34]. The scale variations for $\sigma _i^{(1)}$ and $\sigma _i^{(2)}$ are evaluated to be within 1–25%. We assumed that the scale uncertainty is 100% correlated among the terms $\sigma _{\mathrm {SM}}$, $\sigma _i^{(1)}$, and $\sigma _i^{(2)}$.

### Exclusion limits on the ${\hbox {C}}_{\mathrm{G}}$ effective coupling

In order to constrain the ${\hbox {C}}_{\mathrm{G}}$ coupling, the effect on the $\mathrm{t}{\bar{\mathrm{t}}}$ rate in various (*n*-jets,*m*-tags) categories is considered. The impact of the difference between the kinematic distributions of $\mathrm{t}{\bar{\mathrm{t}}}$ events from the $O_{\mathrm{G}}$ interaction and from the SM interactions on the acceptance is evaluated to be 3% for ${\hbox {C}}_{\mathrm{G}}\sim 1$. This uncertainty is considered only for the ${\hbox {C}}_{\mathrm{G}}$ coupling since the top $p_{\mathrm {T}}$  spectrum is affected considerably by this operator, while other operators lead to a $p_{\mathrm {T}}$  spectrum similar to the SM prediction for unconstrained values of the probed Wilson coefficients. The fit is performed simultaneously on the observed event yields in the categories presented in Fig. 2 in the (1-jet, 1-tag), (2-jets, 1-tag), and ($\ge$2-jets, 2-tags) categories for the $\mathrm{e}\mathrm{e}$, $\mathrm{e}\upmu$, and $\upmu \upmu$ channels. In addition, the (1-jet, 0-tag) category is included only for the $\mathrm{e}\upmu$ channel. The main limiting factor on the constraints in the ${\hbox {C}}_{\mathrm{G}}$ coupling is the uncertainty in the signal acceptance found after maximizing the likelihood, followed by uncertainties in the integrated luminosity calibration and the trigger scale factor.

The results of the fit for the individual channels and for all channels combined are listed in the first row of Table 5.

The results of the likelihood scans of the ${\hbox {C}}_{\mathrm{G}}$ coupling are shown in Fig. 5 (upper left plot). The likelihood scan result of the nominal fit, in which the nominal values of $\sigma _{\mathrm {SM}}$, $\sigma _i^{(1)}$, and $\sigma _i^{(2)}$ terms are assumed, is shown as the thick curve. The thin dashed curves are the results of the fit to the observed data when the assumed values of the $\sigma _{\mathrm {SM}}$, $\sigma _i^{(1)}$, and $\sigma _i^{(2)}$ terms are varied due to the scale and PDF uncertainties. As a second-order parametrization, given by Eq. 6 is used to fit the data, the resulting likelihood function could have two minima, as can be seen in some of the plots in Fig. 5.

### Exclusion limits on the ${\hbox {C}}_{\mathrm{t}\mathrm{G}}$, ${\hbox {C}}_{\phi \mathrm{q}}^{(3)}$, and ${\hbox {C}}_{\mathrm{t}\mathrm{W}}$ effective couplings

In order to set limits on the effective couplings ${\hbox {C}}_{\mathrm{t}\mathrm{G}}$, ${\hbox {C}}_{\phi \mathrm{q}}^{(3)}$, and ${\hbox {C}}_{\mathrm{t}\mathrm{W}}$, we utilize the NN output distributions for both data and MC expectation in the (1-jet, 1-tag) and (2-jets, 1-tag) regions and event yields in the ($\ge$2-jets, 2-tags) region for the three dilepton channels. The inclusion of the ($\ge$2-jets, 2-tags) and (2-jets, 1-tag) categories provides a constraint of the normalization and systematic uncertainties in the $\mathrm{t}{\bar{\mathrm{t}}}$ background. In addition, the (1-jet, 0-tag) category is included for the $\mathrm{e}\upmu$ channel to increase the signal sensitivity. The results of the likelihood scans of the ${\hbox {C}}_{\mathrm{t}\mathrm{G}}$, ${\hbox {C}}_{\phi \mathrm{q}}^{(3)}$, and ${\hbox {C}}_{\mathrm{t}\mathrm{W}}$ Wilson coefficients are shown in Fig. 5 for the combination of all channels. The inclusion of the ${\hbox {C}}_{\mathrm{t}\mathrm{G}}$ coupling to the tW process tightens the 2 standard deviations band by 7%. The results for the individual channels, and the combined results are listed in Table 5 (second, third, and fourth rows). The three main sources of uncertainty that affect the interval determination are uncertainties in the DY estimation, integrated luminosity, and lepton identification scale factors for ${\hbox {C}}_{\mathrm{t}\mathrm{G}}$; jet energy scale, tt and tW interference at NLO, and statistical uncertainty in MC samples for ${\hbox {C}}_{\phi \mathrm{q}}^{(3)}$; statistical uncertainty in data, jet energy scale, and the powheg matching method for ${\hbox {C}}_{\mathrm{t}\mathrm{W}}$ effective couplings.

### Exclusion limits on the ${\hbox {C}}_{\mathrm{u}\mathrm{G}}$ and ${\hbox {C}}_{\mathrm{c}\mathrm{G}}$ effective couplings

Since the $\mathrm{t}\mathrm{W}$ production via FCNC interactions does not interfere with the SM $\mathrm{t}\mathrm{W}$ process (with the assumption of $|\text {V}_{\mathrm{t}\mathrm{d}} | = |\text {V}_{\mathrm{t}\mathrm{s}} | = 0$), the FCNC signal sample is used to set upper bounds on the related Wilson coefficients. Events with exactly one $\mathrm{b}$-tagged jet are included in the limit setting procedure with no requirement on the number of light-flavor jets (*n*-jets, 1-tag). The observed (median expected) $95\%$ confidence level ($\text {CL}$) upper limits on the product of cross section times branching fractions $\sigma (\mathrm{p}\mathrm{p}\rightarrow \mathrm{t}\mathrm{W})\mathcal {B}(\mathrm{W}\rightarrow \ell \upnu )^2$ for the ${\hbox {C}}_{\mathrm{u}\mathrm{G}}$ and ${\hbox {C}}_{\mathrm{c}\mathrm{G}}$ FCNC signals for the combination of the $\mathrm{e}\mathrm{e}$, $\upmu \upmu$, and $\mathrm{e}\upmu$ channels are found to be 0.11 (0.20) pb and 0.13 (0.26) pb, respectively. These results are used to calculate upper limits on the Wilson coefficients ${\hbox {C}}_{\mathrm{u}\mathrm{G}}$, ${\hbox {C}}_{\mathrm{c}\mathrm{G}}$, and on the branching fractions $\mathcal {B}(\mathrm{t}\rightarrow \mathrm{u}\mathrm{g})$ and $\mathcal {B}(\mathrm{t}\rightarrow \mathrm{c}\mathrm{g})$. The limits on the ${\hbox {C}}_{\mathrm{u}\mathrm{G}}$ and ${\hbox {C}}_{\mathrm{c}\mathrm{G}}$ couplings are summarized in the last two rows of Table 5, and correspond to the observed (expected) limits on $\mathcal {B}(\mathrm{t}\rightarrow \mathrm{u}\mathrm{g})<$ 0.12 (0.22)% and $\mathcal {B}(\mathrm{t}\rightarrow \mathrm{c}\mathrm{g})<$ 0.53 (1.05)% at $95\% \, \text {CL}$. The statistical uncertainty in data is the dominant source of uncertainty affecting the limits on the FCNC couplings. The second and third most important uncertainties originate from $\mathrm{t}{\bar{\mathrm{t}}}$ and $\mathrm{t}\mathrm{W}$ interferences at NLO and FSR in $\mathrm{t}{\bar{\mathrm{t}}}$ events.

**Fig. 6:**
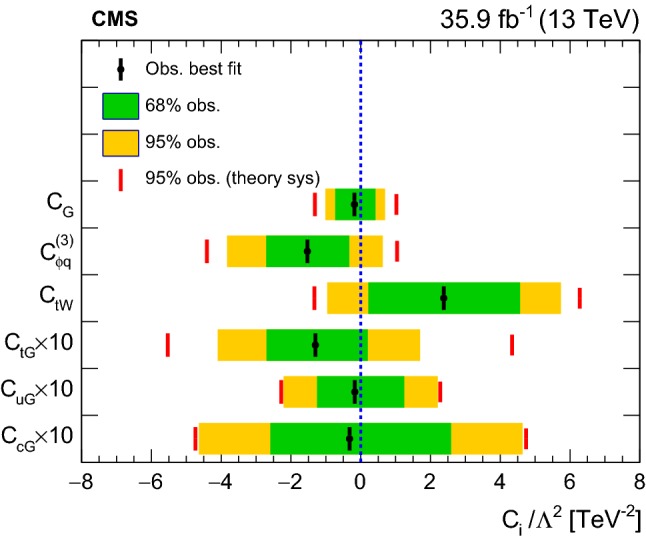
Observed best fits together with one and two standard deviation bounds on the top quark effective couplings. The dashed line shows the SM expectation and the vertical lines indicate the $95\% \, \text {CL}$ bounds including the theoretical uncertainties

The observed best fit together with one and two standard deviation bounds on the six Wilson coefficients, ${\hbox {C}}_{\phi \mathrm{q}}^{(3)}$, ${\hbox {C}}_{\mathrm{t}\mathrm{W}}$, ${\hbox {C}}_{\mathrm{t}\mathrm{G}}$, ${\hbox {C}}_{\mathrm{G}}$, ${\hbox {C}}_{\mathrm{u}\mathrm{G}}$, and ${\hbox {C}}_{\mathrm{c}\mathrm{G}}$, obtained from the combination of all channels are shown in Fig. 6. Table 6 summarizes the effect of the most important uncertainty sources on the observed allowed intervals.

**Table 5:** Summary of the observed and expected allowed intervals on the effective couplings obtained in the $\mathrm{e}\mathrm{e}$, $\mathrm{e}\upmu$, and $\upmu \upmu$ channels, and all channels combined. All sources of systematic uncertainty, described in Sect. 5, are taken into account with the exception of the uncertainties on the SM cross section predictions for the tt and tW processes

Effective coupling	Channel	Observed [${\hbox {TeV}}^{-2}$]			Expected [${\hbox {TeV}}^{-2}$]		
		Best fit	[68% CI]	[95% CI]	Best fit	[68% CI]	[95% CI]
$${\hbox {C}}_{\mathrm{G}} / {\varLambda }^2$$	$$\mathrm{e}\mathrm{e}$$	$$-0.14 $$	$$ [-0.82 , 0.51]$$	$$[-1.14 , 0.83]$$	0.00	$$ [-0.90 , 0.59]$$	$$[-1.20 , 0.88]$$
	$$\mathrm{e}\upmu $$	$$-0.18 $$	$$ [-0.73 , 0.42]$$	$$[-1.01 , 0.70]$$	0.00	$$ [-0.82 , 0.51]$$	$$[-1.08 , 0.77]$$
	$$\upmu \upmu $$	$$-0.14 $$	$$ [-0.75 , 0.44]$$	$$[-1.06 , 0.75]$$	0.00	$$ [-0.88 , 0.57]$$	$$[-1.16 , 0.85]$$
	Combined	$$-0.18 $$	$$ [-0.73 , 0.42]$$	$$[-1.01 , 0.70]$$	0.00	$$ [-0.82 , 0.51]$$	$$[-1.07 , 0.76]$$
$${\hbox {C}}_{\phi \mathrm{q}}^{(3)} / {\varLambda }^2$$	$$\mathrm{e}\mathrm{e}$$	1.12	$$ [-1.18 , 2.89]$$	$$[-4.03 , 4.37]$$	0.00	$$ [-2.53 , 1.74]$$	$$[-6.40 , 3.27]$$
	$$\mathrm{e}\upmu $$	$$-0.70 $$	$$ [-2.16 , 0.59]$$	$$[-3.74 , 1.61]$$	0.00	$$ [-1.34 , 1.12]$$	$$[-2.57 , 2.15]$$
	$$\upmu \upmu $$	1.13	$$ [-0.87 , 2.86]$$	$$[-3.58 , 4.46]$$	0.00	$$ [-2.20 , 1.92]$$	$$[-4.68 , 3.66]$$
	Combined	$$-1.52 $$	$$ [-2.71 , -0.33]$$	$$[-3.82 , 0.63]$$	0.00	$$ [-1.05 , 0.88]$$	$$[-2.04 , 1.63]$$
$${\hbox {C}}_{\mathrm{t}\mathrm{W}} / {\varLambda }^2$$	$$\mathrm{e}\mathrm{e}$$	6.18	$$ [-3.02 , 7.81]$$	$$[-4.16 , 8.95]$$	0.00	$$ [-2.02 , 6.81]$$	$$[-3.33 , 8.12]$$
	$$\mathrm{e}\upmu $$	1.64	$$ [-0.80 , 5.59]$$	$$[-1.89 , 6.68]$$	0.00	$$ [-1.40 , 6.19]$$	$$[-2.39 , 7.18]$$
	$$\upmu \upmu $$	$$-1.40 $$	$$ [-3.00 , 7.79]$$	$$[-4.23 , 9.01]$$	0.00	$$ [-2.18 , 6.97]$$	$$[-3.63 , 8.42]$$
	Combined	2.38	[0.22, 4.57]	$$[-0.96 , 5.74]$$	0.00	$$ [-1.14 , 5.93]$$	$$[-1.91 , 6.70]$$
$${\hbox {C}}_{\mathrm{t}\mathrm{G}} / {\varLambda }^2$$	$$\mathrm{e}\mathrm{e}$$	$$-0.19 $$	$$ [-0.40 , 0.02]$$	$$[-0.65 , 0.22]$$	0.00	$$ [-0.22 , 0.21]$$	$$[-0.44 , 0.41]$$
	$$\mathrm{e}\upmu $$	$$-0.03 $$	$$ [-0.19 , 0.11]$$	$$[-0.34 , 0.27]$$	0.00	$$ [-0.17 , 0.15]$$	$$[-0.34 , 0.29]$$
	$$\upmu \upmu $$	$$-0.15 $$	$$ [-0.34 , 0.02]$$	$$[-0.53 , 0.19]$$	0.00	$$ [-0.19 , 0.18]$$	$$[-0.40 , 0.35]$$
	Combined	$$-0.13 $$	$$ [-0.27 , 0.02]$$	$$[-0.41 , 0.17]$$	0.00	$$ [-0.15 , 0.14]$$	$$[-0.30 , 0.28]$$
$${\hbox {C}}_{\mathrm{u}\mathrm{G}} / {\varLambda }^2$$	$$\mathrm{e}\mathrm{e}$$	$$-0.017 $$	$$ [-0.22 , 0.22]$$	$$[-0.37 , 0.37]$$	0.00	$$ [-0.29 , 0.29]$$	$$[-0.42 , 0.42]$$
	$$\mathrm{e}\upmu $$	$$-0.017 $$	$$ [-0.17 , 0.17]$$	$$[-0.29 , 0.29]$$	0.00	$$ [-0.26 , 0.26]$$	$$[-0.38 , 0.38]$$
	$$\upmu \upmu $$	$$-0.017 $$	$$ [-0.17 , 0.17]$$	$$[-0.29 , 0.29]$$	0.00	$$ [-0.27 , 0.27]$$	$$[-0.38 , 0.38]$$
	Combined	$$-0.017 $$	$$ [-0.13 , 0.13]$$	$$[-0.22 , 0.22]$$	0.00	$$ [-0.21 , 0.21]$$	$$[-0.30 , 0.30]$$
$${\hbox {C}}_{\mathrm{c}\mathrm{G}} / {\varLambda }^2$$	$$\mathrm{e}\mathrm{e}$$	$$-0.032 $$	$$ [-0.47 , 0.47]$$	$$[-0.78 , 0.78]$$	0.00	$$ [-0.63 , 0.63]$$	$$[-0.92 , 0.92]$$
	$$\mathrm{e}\upmu $$	$$-0.032 $$	$$ [-0.34 , 0.34]$$	$$[-0.60 , 0.60]$$	0.00	$$ [-0.56 , 0.56]$$	$$[-0.81 , 0.81]$$
	$$\upmu \upmu $$	$$-0.032 $$	$$ [-0.36 , 0.36]$$	$$[-0.63 , 0.63]$$	0.00	$$ [-0.58 , 0.58]$$	$$[-0.84 , 0.84]$$
	Combined	$$-0.032 $$	$$ [-0.26 , 0.26]$$	$$[-0.46 , 0.46]$$	0.00	$$ [-0.46 , 0.46]$$	$$[-0.65 , 0.65]$$

**Table 6:** Estimation of the effect of the most important uncertainty sources on the observed allowed intervals of in the fit

Uncertainty	${\hbox {C}}_{\mathrm{G}}$ (%)	${\hbox {C}}_{\phi \mathrm{q}}^{(3)}$ (%)	${\hbox {C}}_{\mathrm{t}\mathrm{W}}$ (%)	${\hbox {C}}_{\mathrm{t}\mathrm{G}}$ (%)	${\hbox {C}}_{\mathrm{u}\mathrm{G}}$ (%)	${\hbox {C}}_{\mathrm{c}\mathrm{G}}$ (%)
Trigger	10.2	2.3	7.0	2.9	1.7	2.5
Lepton ident./isolation	7.4	1.1	1.2	23.0	<1	<1
Jet energy scale	<1	25.0	17.8	4.9	<1	<1
$\mathrm{t}\mathrm{W}$ DS/DR	<1	24.2	4.4	3.0	7.6	7.8
ME/PS matching	<1	4.9	9.9	1.2	<1	<1
ISR scale	<1	5.0	5.6	<1	<1	<1
FSR scale	5.8	4.4	4.0	10.2	<1	<1
DY background	<1	7.5	5.5	21.5	<1	<1
Nonprompt background	<1	1.4	5.8	<1	<1	<1
Integrated luminosity	13.1	<1	1.1	18.8	<1	<1
Statistical	5.8	2.3	23.7	<1	72.6	73.6
MC statistical	<1	12.1	3.7	5.2	2.9	2.5

## Summary

A search for new physics in top quark interactions is performed using $\mathrm{t}{\bar{\mathrm{t}}}$ and $\mathrm{t}\mathrm{W}$ events in dilepton final states. The analysis is based on data collected in $\mathrm{p}\mathrm{p}$ collisions at $13\,\text {TeV}$ by the CMS detector in 2016, corresponding to an integrated luminosity of $35.9{\,\text {fb}^{-1}}$. No significant excess above the standard model background expectation is observed. For the first time, both $\mathrm{t}{\bar{\mathrm{t}}}$ and $\mathrm{t}\mathrm{W}$ production are used simultaneously in a model independent search for effective couplings. The six effective couplings, ${\hbox {C}}_{\mathrm{G}}$, ${\hbox {C}}_{\mathrm{t}\mathrm{G}}$, ${\hbox {C}}_{\mathrm{t}\mathrm{W}}$, ${\hbox {C}}_{\phi \mathrm{q}}^{(3)}$, ${\hbox {C}}_{\mathrm{u}\mathrm{G}}$, and ${\hbox {C}}_{\mathrm{c}\mathrm{G}}$ are constrained using a dedicated multivariate analysis. The constraints presented, obtained by considering one operator at a time, are a useful first step toward more global approaches.

## Data Availability

This manuscript has no associated data or the data will not be deposited. [Authors’ comment: Release and preservation of data used by the CMS Collaboration as the basis for publications is guided by the CMS policy as written in its document "CMS data preservation, reuse and open access policy" (https://cms-docdb.cern.ch/cgibin/PublicDocDB/RetrieveFile?docid=6032&filename=CMSDataPolicyV1.2.pdf&version=2 ).]
